# Genomic and Transcriptomic Landscapes of MEN1-Wild-Type Low-Grade Metastatic Pancreatic NETs Uncover Key Oncogenic Drivers and Targetable Pathways

**DOI:** 10.7150/ijbs.134269

**Published:** 2026-09-03

**Authors:** Md. Hafiz Uddin, Zaid Mahdi, Irfana Muqbil, Brendon R. Herring, Bart Rose, Husain Y. Khan, Yiwei Li, Amro Aboukameel, Sahar F. Bannoura, Hugo Jimenez, Allan M. Johansen, Mohammed Najeeb Al-Hallak, Ibrahim Azar, Amr Mohamed, Tarik Hadid, Nitin Vaishampayan, Yang Shi, Yin Wan, Vy Ong, Gregory Dyson, Rafic Beydoun, Miguel Tobon, Eliza W. Beal, Herbert Chen, Anthony F. Shields, Philip A. Philip, Jennifer Beebe-Dimmer, Ramzi M. Mohammad, Boris C. Pasche, Bassel F. El-Rayes, Asfar S. Azmi

**Affiliations:** 1 Karmanos Cancer Institute, Wayne State University, Detroit, MI, USA.; 2 Emory Winship Cancer Institute, Atlanta, GA, USA.; 3 Lawrence Technological University, Southfield, MI, USA.; 4 O'Neal Comprehensive Cancer Center, University of Alabama at Birmingham, Birmingham, AL, USA.; 5 Department of Cancer Biology, Wake Forest University, Winston-Salem, NC, USA.; 6 Trinity Health Oakland, Pontiac, MI, USA.; 7 UH Seidman Cancer Center, University Hospitals, Case Western Reserve University, Cleveland, OH, USA.; 8 Department of Pathology, Wayne State University School of Medicine, Wayne State University, Detroit, MI, USA.; 9 Department of Oncology and Pharmacology, Henry Ford Cancer Institute, Detroit, MI, USA.; 10 Division of Medical Oncology, Moores Cancer Center, UC San Diego, La Jolla, CA, 92037, USA.

**Keywords:** pancreatic neuroendocrine tumors, genomic and transcriptomic characterization, low grade, metastasis, mutation, therapeutic candidates

## Abstract

Sporadic pancreatic neuroendocrine tumors (pNETs) with wild-type MEN1 represent a major yet largely ignored subset whose biology and metastatic potential remain poorly understood. Because metastasis can occur despite low histologic grade and modest mutational burden, we hypothesized that metastatic competence in MEN1-wild-type pNETs reflects quantitative reinforcement of shared oncogenic pathways rather than distinct mutational processes. We profiled 75 primary WHO G1/G2 pNETs by whole-exome and RNA sequencing, including 25% with lymph node and/or liver metastasis, and integrated genomic and transcriptomic data to connect pathway lesions with expression state. Metastatic tumors showed a slight increase in mutation frequency but conserved base-substitution spectra relative to non-metastatic cases, and adverse clinicopathologic features were enriched in Grade 2 disease. Aggregating alterations to pathways revealed broad convergence on canonical networks, with transcriptomic analyses demonstrating cohort-wide enrichment of Calcium, WNT, and KRAS/PI3K-AKT programs in metastasis. Intersection of significantly mutated genes with differentially expressed genes identified a focused 29-gene overlap, including RYR1 and ZNF273, that marks these convergent axes and distinguishes metastatic from non-metastatic tumors. Gene set enrichment confirmed preferential activation of Calcium, WNT, and PI3K-AKT signaling in metastatic tumors, consistent with a network-intensity model of progression. Finally, upstream-regulator analysis (iPathwayGuide) and gene-centric perturbation mapping (Gene2Drug) nominated candidate targeted and repurposable agents predicted to reverse the metastatic expression phenotype and flagged drugs unlikely to provide benefit, yielding a prioritized, testable therapeutic shortlist which includes fasudil and spaglumic acid. Convergent, domain-specific mutational patterns in highly mutated genes such as ZNF273 and CLCA1 define a molecular signature that could stratify metastatic risk in low-grade pNETs. Functional validation identified ZNF273 and RYR1 as candidate effectors of metastatic fitness in MEN1-wild-type pNETs, as siRNA-mediated silencing reduced cell viability, clonogenicity, spheroid growth, and migration in BON1 and QGP1 models. Predicted compounds showed *in vitro* activity, with doxorubicin exhibiting the strongest cytotoxic effect, supporting a therapeutically tractable KRAB-ZNF/calcium signaling axis in metastatic pNETs. Collectively, our data reframe metastasis in MEN1-wild-type low-grade pNETs as a property of pathway state rather than mutation quantity and provide a translational blueprint for biomarker-guided therapy development focused on Calcium, WNT, and KRAS/PI3K hubs.

## Introduction

Neuroendocrine tumors (NETs) arise from the hormone-producing cells of various organs throughout the body and include pancreatic NETs (pNETs), medullary thyroid cancer, gastrointestinal (GI) NETs, bronchopulmonary NETs, and paraganglioma/pheochromocytoma^1^. Over 50% of patients with pNETs develop isolated hepatic metastases^1^. According to the American Cancer Society's estimate, in the United States over 4,000 individuals were diagnosed with pNETs in 2020 (ACS Statistics)^2^. Overall survival for pNETs can be relatively long compared to other cancers, leading to a higher number of cases of this disease at any given time^3^, ^4^. pNETs are heterogeneous neoplasms with rising incidence and substantial clinical variability, ranging from indolent lesions to tumors that metastasize despite low histologic grade, thus the management of pNETs remains clinically challenging^2^. Improvements in the identification of actionable oncogenic drivers or systemic treatments for this growing patient population have been at best modest in recent decades. Therefore, pNETs remain a significant unmet clinical problem and in urgent need of newer biomarkers as well as more effective therapeutics.

pNETs can result from heritable genetic or somatic non-familial mutations^5^, ^6^. Earlier, large-scale genomic studies revealed that loss of Multiple Endocrine Neoplasia Type 1 (MEN1), Death-Domain-Associated protein (DAXX), and α Thalassemia/mental Retardation Syndrome X-linked (ATRX) genes as the major genetic aberrations in pNETs^7^. Additionally, the hyperactivation of PI3K-Akt-mTOR through loss of tumor suppressor PTEN has been well documented as a main driver in pNETs^8^, ^9^. Molecular studies over the past decade have defined recurrent alterations in MEN1, DAXX, and ATRX, and frequent perturbations of PI3K-mTOR signaling, establishing a canonical framework for tumorigenesis and motivating targeted therapy trials^7^, ^10^-^13^. These genomic advances translated into clinically meaningful gains with agents such as everolimus^14^ and sunitinib, which prolong progression-free survival in advanced disease^12^, yet durable control and robust biomarkers remain limited^11^, ^15^, ^16^. More recent multi-omic and population-level analyses underscore that tumor evolution and outcome are not fully explained by the presence or absence of MEN1/DAXX/ATRX lesions alone, and that broader network-level perturbations likely shape malignant potential and therapeutic response^17^-^19^. Within this landscape, MEN1-wild type disease constitutes a major and under-characterized subset of sporadic pNETs in which the determinants of metastatic behavior and drug sensitivity remain unclear.

Despite progress in cataloging mutations, key biological/clinical questions persist^20^. Our mechanistic understanding was derived from MEN1-mutation enriched cohorts, leaving MEN1-wild type pNETs relatively unresolved^18^, ^21^, ^22^. While PI3K-mTOR axis activity is common, it incompletely predicts metastatic risk or treatment benefit, implying that additional modulators of aggressiveness are involved^17^, ^23^. In pNETs, low-to-moderate tumor mutational burden does not reliably stratify outcomes, suggesting that metastasis may reflect reinforcement of shared oncogenic pathways rather than qualitative shifts in mutational processes^24^. To address these gaps, we have excluded high-grade and MEN1-mutated pNETs and assembled and profiled a rigorously curated cohort of MEN1-wild type WHO G1/G2 grade (hereafter low-grade) pNETs with or without metastatic potential and used matched whole-exome and RNA sequencing data integration to connect genomic lesions with transcriptional state. The current study provides a translational bridge by applying iPathwayGuide and Gene2Drug to nominate candidate therapeutics^25^-^28^. Our guiding hypothesis was that metastatic behavior reflects convergence on a limited set of oncogenic pathways.

## Methods

### Collection, processing and sequencing of patient samples

Under an IRB-approved protocol (STUDY00001739), a total of 104 Formalin-Fixed, Paraffin-Embedded (FFPE) tissues (98 low-grade pancreatic neuroendocrine tumor (pNET) and six normal pancreatic) from the Emory University tissue biobank were sectioned and the genomic DNA was extracted using QIAamp DNA FFPE Tissue Kit (Qiagen, Germany). Sex was considered as a biological variable and average age of the patients was 55.83 years. **Figure 1A** shows tissue selection schema.

### Collection of pathological records

Primary tumor characteristics of the pNET patients were obtained from the histo- and cytopathology registry at Emory University. Data collected included patient gender, WHO-defined tumor differentiation grade (Grade 1 or Grade 2), proliferation index (Ki-67), and evidence of invasion or metastasis (supplementary method).

### Whole exome sequencing

For whole exome sequencing (WES), DNA samples from pNET and normal tissues were fragmented by using sonication and subjected to library construction in blinded fashion (supplementary method).

### Mutational signature analysis

The Genome Analysis Toolkit (GATK) was utilized to identify the genomic variants in the WES data (supplementary method).

### RNA-sequencing

Total RNA was extracted from pNET and normal tissues using the miRNeasy mini kit from Qiagen. The primary pNETs and pancreatic normal FFPE (Formalin-Fixed, Paraffin-Embedded) tissues were sectioned, and the total RNA was extracted following manufacturer's instruction (Qiagen, Germany). Total RNA-sequencing was conducted by LC Sciences (supplementary method). Expression levels of mRNAs were quantified using StringTie^29^ based on fragments per kilobase of transcript per million mapped reads (FPKM).

### Inventory of clinically actionable putative therapeutic targets against somatic alterations

We prioritized candidate therapies by integrating pathway-level upstream regulator inference (iPathwayGuide, Advaita Bio; AKB v18.1, 2025 release) with gene-centric drug perturbation profiling (Gene2Drug, accessed 2025) using the same tumor set (supplementary method).

### *In vitro* assays

We used pNET cell lines BON1 and QGP1 and neuroendocrine carcinoma cell line NECT2 for* in vitro* validation studies. Other assays include 2D/3D cell viability, colony formation, spheroid formation, trans-well migration, RT-qPCR, western blotting, intracellular calcium flux assay, immunohistochemistry, immunofluorescence using standard procedure and detailed in the supplementary method.

### *In vivo* cell line-derived xenograft (CDx) models

Both BON1 and QGP1 CDx models were utilized to determine the efficacy of prioritized drugs Fasudil and Harmol (supplementary method). *In vivo* studies were performed according to Wayne State University's Institutional Animal Care and Use Committee (IACUC) approved protocol (# 25-02-7587) and guidelines.

### Statistical analysis

All analyses were performed on primary low-grade pNETs with matched WES and RNA-seq. For RNA-Seq, differentially expressed genes were assessed with the R Bioconductor package edgeR with the thresholds of absolute value of log2 (fold change) > 1 and FDR < 0.05 (supplementary method).

## Results

### Clinicopathologic stratification of low-grade pNETs for integrated genomic and transcriptomic analysis

To investigate molecular determinants of metastatic behavior, the primary tumors were subsequently stratified into two major categories according to their metastatic outcome (**Figure 1B**) including low-grade non-metastatic and metastatic pNETs. Both groups underwent a unified workflow, including DNA and RNA extraction from FFPE blocks, WES and RNA-seq, and integrated genomic-transcriptomic analysis for candidate target identification. Among the primary tumors, 7 Grade-1 and 12 Grade-2 pNETs were with metastatic disease (**Figure 1A**), providing a clinically annotated set of cases for comparative analyses with non-metastatic counterparts. The demographic and pathological characteristics of the sequenced cohort are summarized in **Figures 1C-1D** and **Table 1**. Age at diagnosis displayed a wide distribution in both sexes, with overlapping ranges and comparable median ages for male and female patients (**Figure 1C**), indicating that metastatic propensity in this cohort is not driven by major age or sex imbalances. In contrast, adverse histopathologic features were enriched among Grade 2 tumors. Grade 2 tumors exhibited substantially higher rates of lympho-vascular invasion (LVI), peri-neural invasion (PNI), and metastasis (METs) compared to Grade 1 (30-37% positive vs. 17-21% in Grade 1; **Figure 1D**). Collectively, these data define a well-curated set of primary low-grade pNETs with linked clinical, histologic, and multi-omic profiles, and underscore the association between metastatic potential and genomic/transcriptomic characteristics.

### Comprehensive landscape of somatic mutations in low-grade pNETs reveals conserved mutational signatures across metastatic states

The global mutational landscape of primary low-grade pNETs was next examined by ordering tumors according to metastatic outcome and histologic grade (**Figure 2**). The overall mutation burden was moderate, with a small subset of outliers, and most cases harboring a relatively low number of mutations (median number of mutations per patient: 1082, range: 10 - 12,800) (**Figure 2A**). The exonic TMB paralleled the distribution of overall mutation burden (**Figure 2C**), with a median of 8.43 and range of 0.07 - 109 mutations per megabase (Mb). However, the median exonic TMBs calculated as mutations per Mb were slightly higher in pNETs with metastatic status (median: 8.01, range: 0.08-109 for non-metastatic; median: 10.90, range: 0.07-106.28 for metastatic). Note that the exonic TMBs in our cohort were higher than those reported in primary pNETs analyzed using whole-genome sequencing (median: 0.82 mutations/Mb, range: 0.04-4.56 mutations/Mb)^9^, but were within the broader range reported in a cohort comprising primary and metastatic pNETs using a targeted cancer-gene sequencing panel (median: 4.7 mutations/MB, range: 0.8-266.4 mutations/Mb)^30^. Inspection of mutation annotations showed moderate exonic variants of total TMB, whereas the majority of variants resided in non-coding regions (**Figure 2B**). Within coding regions, nonsynonymous single-nucleotide variants (SNVs) and frameshift or non-frameshift insertions/deletions represented the dominant functional classes (**Figure 2D**). The base-substitution spectra revealed a pyrimidine mutation pattern dominated by C>T transitions, with additional contributions from T>C and C>A changes (**Figure 2E**). This six-class SNV profile was highly consistent across the cohort and did not show obvious qualitative shifts between metastatic and non-metastatic tumors. When genomic features were related to clinicopathologic parameters (**Figures 2F-2I**), metastatic cases were clustered toward higher histologic grades and higher Ki-67 percentages. Collectively, these data indicate that while classical clinicopathologic markers (grade, Ki-67, and site of spread) stratify aggressive behavior, metastatic propensity in low-grade pNETs is not simply explained by gross differences in mutational load or base-substitution patterns, motivating deeper pathway-level analyses.

### Recurrently mutated genes and canonical cancer drivers in non-metastatic and metastatic pNETs

To resolve gene-level distinctions between indolent and aggressive primary pNETs, we systematically mapped somatic alterations across the cohort using oncoprint visualizations of the 50 most recurrently mutated genes together with seven canonical cancer-driver genes (**Figure 3** and **Supplementary Figure 1**). All tumors carried at least one coding alteration within this gene set, highlighting a pervasive yet heterogeneous mutational landscape. Samples arranged by decreasing TMB revealed that missense variants comprised the dominant class of alterations, followed by frameshift and nonsense mutations, with multi-hit events concentrated in the most highly mutated tumors. In non-metastatic pNETs, mutations were broadly distributed across large structural and membrane-associated genes (e.g., MUC17, TTN, and AHNAK) and numerous zinc-finger transcription factors (including ZNF729, ZNF208, and ZNF9), many of which exhibited multi-hit patterns (**Figure 3A**). Metastatic tumors displayed a qualitatively similar mutational repertoire with mucin genes (MUC17, MUC16, MUC12), cytoskeletal and scaffolding genes (AHNAK, TTN), and multiple zinc-finger transcription factors (ZNF9, ZNF43, and ZNF208) dominating the most frequently altered loci and frequently exhibiting multi-hit events in high-TMB tumors (**Figure 3B**). Canonical drivers in chromatin remodeling and PI3K-mTOR signaling were similarly recurrently mutated, indicating shared pathway disruption across disease states. Consistent with this pattern, bubble scatterplot analysis of mutation prevalence showed most genes aligning near the diagonal, reflecting broad parity across groups, while a limited subset deviated above the line, indicating relatively higher prevalence in metastatic tumors (**Figure 3C**). Together, these results support a conserved mutational backbone in low-grade pNETs centered on mucin/structural genes and zinc-finger transcription factors with selective frequency shifts in a small subset of genes in metastatic disease, suggesting that metastatic divergence likely emerges from additional regulatory layers beyond gene-level mutational rewiring.

### Convergent disruption of canonical oncogenic pathways and enriched proliferative programs in metastatic pNETs

We next examined how the somatic mutational landscape in pNETs converges on established oncogenic signaling pathways. To assess such biological relevance of the observed mutations, we performed pathway enrichment analysis and observed enrichment of RTK-RAS signaling, WNT/β-catenin, NOTCH, Hippo, cell cycle regulation, TGF-β signaling, PI3K/mTOR, Myc, and p53 pathways (**Figure 3D-3E**). Notably, the mutated genes in the RTK-RAS, WNT, NOTCH, and Hippo pathways were found to be activated in pancreatic cancer tissues^31^, suggesting that these alterations may contribute to pNET tumorigenesis through shared oncogenic mechanisms. Although a subset of the many genes annotated to each pathway were mutated, these alterations collectively affected the pathway deregulation rather than single-gene lesions which could be a defining feature of pNET genomics.

Binary mutation maps across RTK-RAS, NOTCH, WNT, and Hippo signaling revealed pronounced inter-tumoral heterogeneity and pathway-level convergence (**Figure 3E**). Within each pathway, somatic alterations affected diverse receptors, core transducers, and transcriptional effectors. Mutation in core canonical drivers (red) and essential, often druggable, signaling nodes (blue) indicates that distinct mutational combinations perturb the same signaling axis. Hypermutated tumors frequently exhibited multi-hit disruption within individual pathways, whereas lower-burden tumors more commonly carried single-gene lesions. Comparison of metastatic and non-metastatic tumors showed a shared core of mutated genes superimposed on subgroup-specific alterations, with functional enrichment of metastatic mutations in Calcium, PI3K-Akt, WNT and related growth-signaling pathways (**Figure 3F, 3G**) and hallmark proliferative programs including mitotic spindle, E2F targets, and KRAS signaling (**Figure 3H**). NOTCH and WNT signaling pathways are also found to be enriched in grade 2 metastatic pNETs (**Supplementary Figure 2**). Collectively, these findings indicate that heterogeneous gene-level alterations converge on a limited set of canonical oncogenic pathways that may underlie metastatic competence and nominate actionable signaling nodes.

### Transcriptomic reprogramming and metastasis-associated gene expression programs in low-grade pNETs

We first compared the transcriptomes of low-grade pNETs with normal pancreas to define tumor-specific gene expression programs (**Figure 4**). The volcano plot revealed a substantial transcriptional rewiring (**Figure 4A**) where most strongly downregulated genes were the immune and signaling regulators MKNK1-AS1, SOS2, and CXCL10, whereas highly upregulated transcripts included SNTG1, RIMS2 and GPC6 implicated in synaptic signaling, adhesion, and receptor regulation. The differential expression pattern was consistent when compared between non-metastatic and metastatic categories in Grade 1, Grade 2 or all pNETs (**Supplementary Figure 3**). Unsupervised hierarchical clustering of all differentially expressed genes (DEGs) segregated tumors into distinct expression clusters (**Figure 4B**). The annotation tracks demonstrated that metastatic status, WHO grade, and tumor size were non-randomly distributed across the dendrogram. These findings suggest that discrete transcriptomic states underlie a spectrum of biological behavior within histologically low-grade pNETs.

To explore functional consequences, we performed enrichment analyses on DEGs between non-metastatic and metastatic tumors (**Figure 4C**). The top enriched pathways/terms converged on programs related to cell-cycle regulation, DNA replication and repair, extracellular matrix organization, cell-cell/cell-matrix interaction, and cytokine/immune signaling, indicating that metastatic tumors are preferentially enriched for proliferative, matrix-remodeling, and microenvironment-interacting gene sets. Gene-set enrichment analysis on these DEGs between non-metastatic and metastatic tumors using curated KEGG and hallmark signatures further highlighted these differences (**Figure 4D-4E**). Together, these data demonstrate that metastatic competence is associated with a distinct enrichment of gene expression programs supporting proliferation, stress adaptation, and tumor microenvironment crosstalk.

### Integrated genomic-transcriptomic analysis identifies Calcium, WNT, and KRAS/PI3K signaling as convergent axes in low-grade pNETs

To pinpoint alterations that are both genetically and transcriptionally encoded in the same tumors, we integrated somatic mutation and RNA-seq data from the low-grade pNET cohort (**Figure 5**). Intersection of significantly mutated genes with DEGs revealed a relatively small but potentially significant overlap (0.6%, p-value from a permutation test assessing the significance of the overlap: 0.0099) (**Figure 5A**). Thus, while pNETs display broad transcriptional reprogramming, a restricted subset of genes shows concordant DNA- and RNA-level deregulation. The 29 overlapping genes are mutated at variable frequencies across non-metastatic and metastatic tumors including zinc finger protein 273 gene ZNF273, intracellular calcium release channel gene ryanodine receptor 1 (RYR1). Pathway mapping of the overlapping gene set highlighted **Calcium signaling, WNT signaling, and KRAS/PI3K-related programs** as major convergent nodes. In the Calcium signaling pathway, multiple genes were mutated more frequently and expressed differentially in metastatic samples (**Figure 5B**, upper and lower panels respectively), supporting functional perturbation of calcium-dependent signaling in clinically aggressive disease. Calcium signaling along with motor proteins are also highlighted in Grade 2 metastatic pNETs (**Supplementary Figure 4**). A similar pattern was observed for the WNT pathway, efferocytosis, inflammatory response and E2F targets (**Figure 5C** and **Supplementary Figure 5**) genes, suggesting that these signaling dysregulations arise through a combination of genetic lesions and transcriptional remodeling. Besides, several components of Hallmark KRAS signaling DN gene set were mutated at appreciable frequencies in metastatic tumors (**Supplementary Figure 5D**), implying that KRAS-related signaling, although not always driven by classic KRAS mutations, is modulated through alterations in its downstream effector network.

To assess whether these pathways are coordinately enriched at the transcriptomic level in metastatic disease, we performed gene set enrichment analysis (GSEA). Calcium signaling, WNT signaling, and PI3K-AKT signaling were all positively enriched in metastatic pNETs (**Supplementary Figure 5E**) indicating a consistent, cohort-wide shift toward higher pathway activity in metastatic pNETs. We have analyzed mutational patterns for top overlapping genes including ZNF273, RYR1, CLCA1, CEACAM5, MYO3B and observed distinct distributions of mutations both in percentage and types in metastatic compared to non-metastatic pNET samples (**Figure 5D** and **Supplementary Figure 6**). A higher rate of somatic mutation (52.6%) was evident in metastatic pNETs for ZNF273 particularly accumulated in KRAB_A-box domain. Collectively, these integrative analyses demonstrate that only a small fraction of genes are both mutated and differentially expressed and tend to cluster within a limited set of signaling pathways that are active in metastatic tumors. Mutational distribution demonstrates that metastatic pNETs are enriched for structurally diverse and potentially more deleterious mutations. These convergent axes likely represent key molecular circuits underpinning metastatic competence in low-grade pNETs and may provide rational targets for therapeutic intervention or biomarker development.

### *In-silico* drug prioritization reveals candidate targeted and repurposable therapies for metastatic low-grade pNETs

To explore whether the molecular alterations identified in metastatic low-grade pNETs might be pharmacologically tractable, we applied complementary pathway- and gene-centric drug prediction strategies. Upstream regulator analysis using iPathwayGuide, which integrates the direction and magnitude of DEGs with a curated drug-target interaction network, nominated several compounds whose known targets were consistently perturbed in metastatic pNETs. The agents listed in **Figure 5E** (left panel) including Thiram and Ivermectin represent clinically actionable candidates. In contrast, agents like Zeranol may be less likely to provide benefit in metastatic low-grade pNETs and could even be counter-therapeutic (**Figure 5E** right panel). Gene2Drug queried multiple functional databases to identify drugs that reproducibly downregulate expression programs. The software predicted several compounds with strong inhibitory (DOWN) signatures with relatively high enrichment scores, suggesting that their transcriptional footprints oppose the ZNF273 or RYR1-associated expression pattern in metastatic tumors (**Figure 5F** and **Supplementary Figure 7-8**).

### *In vitro* validation demonstrates that ZNF273 and RYR1 promote pNET cell growth, migration, and drug vulnerability

RT-qPCR analysis confirmed that ZNF273 and RYR1 are markedly overexpressed in pNET models along with ZNF184, CEACAM5, -7 relative to human islet control, with both BON1, QGP1, as well as neuroendocrine carcinoma cells NECT2 showing substantially higher baseline transcript abundance (**Figure 6A** and **Supplementary Figure 9A-B**). Among the top downregulated genes we observed differential expressions of SORBS1, CLCA1 in BON1, QGP1 or NECT2 cell lines (**Supplementary Figure 9C**). Efficient target suppression was then achieved by siRNA in both cell lines, as shown by reduced ZNF273 and **RYR1** mRNA levels following gene-specific silencing (**Figure 6B**). Functionally, knockdown of either target significantly impaired short-term cell survival at 72 hours or 5 days, with MTT assays showing decreased viability in BON1 and QGP1 compared with non-transfected or control siRNA-treated cells (**Figure 6C**). Extensive vacuolization was prominent in RYR1 silenced BON1 cells (**Supplementary Figure 10**). This growth-suppressive effect extended to long-term clonogenic assays which demonstrated a marked reduction in colony formation after ZNF273 or RYR1 silencing in both models (**Figure 6D**). Together, these data support a role for ZNF273 and RYR1 in maintaining pNET cell growth and clonogenic fitness.

Consistent with these findings, 3D spheroid assays showed that depletion of ZNF273 or RYR1 reduced spheroid growth/viability in BON1 and QGP1, as reflected by smaller representative spheroids and lower CellTiter-Glo luminescence signals (**Figure 6E**). To demonstrate the impact of ZNF273 and RYR1 silencing on the migratory potential, we performed Boyden chamber migration assays. We observed significantly fewer migrated cells after knockdown of either gene, indicating that both targets also contribute to the migratory phenotype of pNET cells (**Figure 6F** and **Supplementary Figure 10A-B**). To explore therapeutic tractability as described in **Figure 5E**-**F**, predicted compounds were evaluated across micromolar concentration ranges in dose-response assays. All tested predicted drugs including harmol, buspirone, doxorubicin and fasudil showed robust growth inhibition at pharmacologically relevant concentrations in both BON1 and QGP1 cells. Among the drugs, doxorubicin displayed the most pronounced cytotoxic activity (**Figure 6G**). Consistent with this, treatment of QGP1 spheroids with 1 µM doxorubicin for 72 hours decreased spheroid viability compared with vehicle-treated controls, supported by both representative images and ATP-based proliferation assay (**Figure 6H**). Although zoledronic acid was predicted as a non-beneficial drug (**Figure 5E** right panel), we did not observe any growth promoting activity with a wide range of concentrations (**Supplementary Figure 11C**).

### Functional validation of ZNF273 and RYR1 and *in vivo* evaluation of predicted therapeutic agents in pNETs

Immunohistochemical analysis showed higher ZNF273 and RYR1 expression in liver metastatic G1/G2 pNETs (ZNF273: n = 23 vs 9, P = 0.03; RYR1: n = 27 vs 10, P = 0.05) (**Figure 7A-B**). RYR1 silencing altered calcium-channel regulatory genes resulting in lower STIM1:ORAI1 ratios (0.64:1 and 1.05:1 for BON1 and QGP1 respectively) (**Figure 7C**) and reduction of intracellular Ca²⁺ signaling (**Figure 7D**). Predicted drugs such as harmol and spaglumic acid that work via RYR1 also reduced the STIM1:ORAI1 ratios (**Supplementary Figure 12**). ZNF273 protein was expressed in BON1 and QGP1 as well as neuroendocrine carcinoma cell NECT2 and was efficiently depleted by siRNA (**Figure 7E**). RYR1 silencing was evident from immunofluorescence analysis (**Supplementary Figure 13**). The predicted drugs showed target engagement via the reduction of ZNF273, RYR1 or β-Catenin (**Figure 7F**). We have observed robust increase in predicted drug doxorubicin sensitivity upon ZNF273 silencing (**Figure 7G** and **Supplementary Figure 14**). Finally, fasudil and harmol suppressed BON1 and QGP1 xenograft growth and reduced endpoint tumor weights (BON1: *P* = 0.011 and *P* = 0.005; QGP1: *P* = 0.02 and *P* = 0.03, respectively) without marked body-weight loss.

Although exploratory and requiring functional validation, these integrative analyses demonstrate that metastatic low-grade pNETs harbor molecular alterations that map onto existing pharmacologic space. By combining pathway-level upstream regulator analysis with gene-centric perturbation signatures, our data nominate a focused set of candidates and repurposable agents that may be capable of modulating key transcriptional programs in metastatic low-grade pNETs.

## Discussion

Pancreatic neuroendocrine tumors lacking MEN1 mutations comprise a major and under characterized subset. By profiling low-grade primary pNETs with whole-exome and RNA sequencing, including 25 percent with documented metastasis, we close a key gap by showing that metastatic behavior emerges from pathway-level convergence rather than general changes in mutational burden. To our knowledge, this is the first comprehensive study integrating genomic and transcriptomic landscapes of low-grade pNETs to distinguish metastatic from non-metastatic disease. The central advance is that metastatic propensity in these histologically low-grade tumors is not explained by bulk mutational load or shifts in substitution spectra but is constrained by coordinated activation of Calcium, WNT, and KRAS/PI3K pathway programs. These are detectable at the transcriptome level and overlap with a small set of genes that are both mutated and differentially expressed, including ZNF273 and RYR1. Importantly, upstream regulator and gene-centric perturbation analyses nominate tractable candidates predicted to reverse metastatic expression states while flagging agents unlikely to benefit this population, providing a prioritized therapeutic hypothesis for prospective validation. Our data suggest that convergent, domain-specific mutational patterns in frequently mutated genes may constitute a molecular signature capable of stratifying metastatic risk in low-grade pNETs. These findings refine a MEN1 centric model of pNET tumorigenesis by shifting emphasis toward transcriptomic reinforcement of shared oncogenic pathways rather than acquisition of unique mutational processes.

While Chan et al. (2018) and other research groups characterized molecular subtypes defined by MEN1, DAXX, and ATRX mutations, their analyses primarily focused on MEN1-mutant tumors, leaving the MEN1-wild type subset (which accounts for ~60% of pNETs) less explored^17^, ^18^, ^23^. Our data position MEN1-wild type low-grade pNETs as tumors in which metastatic potential arises from quantitative reinforcement of shared oncogenic circuits rather than acquisition of new mutational processes. Clinicopathologic enrichment of adverse features in Grade 2 tumors (**Figure 1D**) and a modest rise in mutation burden in metastatic cases (**Figure 2A**, **2C**) occur without broad changes in base-substitution spectra (**Figure 2E**), indicating conserved mutational mechanisms across disease states. Oncoprint analysis shows substantial overlap in recurrently altered genes between non-metastatic and metastatic tumors, particularly involving chromatin remodelers and PI3K-mTOR components, while frequency skews for a limited subset of genes are evident in metastasis (**Figure 3A-3C**). This pattern reinforces the concept that pathway activation, in addition to mutational quantity, drives metastatic progression. These findings refine earlier genomic observations showing that sporadic pNETs generally harbor low to moderate TMB and recurrent mutations in chromatin-remodeling and PI3K-mTOR pathways^32^, ^33^. Moreover, recent reviews emphasize that PI3K/AKT/mTOR signaling alone does not fully predict metastatic behavior, suggesting the involvement of broader network reinforcement^34^.

Virtually all tumors in our MEN1-wild-type, low-grade pNET cohort, carry alterations in one or more hallmark pathways with convergence on RTK-RAS, WNT, NOTCH, Hippo, PI3K-mTOR, MYC, TP53, TGF-β and NRF2 modules (**Figure 3D, 3E**), while only a small fraction of genes are uniquely mutated in metastases (**Figure 3F, 3G**). Metastases preferentially activate E2F targets, mitotic spindles and KRAS-linked signatures together with matrix-interaction programs (**Figure 4C-4E**). These data align with studies establishing convergent pathway biology in pNETs in which diverse lesions perturb shared axes such as MEN1/DAXX/ATRX and PI3K-mTOR, and extend them by showing that, in MEN1-wild-type disease, metastatic behavior tracks with pathway intensity rather than the appearance of new categories of mutation^7^, ^9^. The transcriptional enrichment of PI3K-mTOR and KRAS-proximal programs in metastases is concordant with the clinical activity of pathway-directed agents such as everolimus and sunitinib in advanced pNETs, supporting a rationale for biomarker-guided intensification or combination strategies in this subgroup^24^, ^35^.

Integrating mutations and RNA profiles from the same MEN1-wild-type low-grade pNETs, we find that only a narrow fraction of genes shows concordant DNA/RNA deregulation (29 of 5,107; 0.6%), yet these concentrate within three reproducible signaling axes including Calcium, WNT, and KRAS/PI3K that are preferentially engaged in metastatic tumors (**Figure 5**). In Calcium signaling, metastatic cases carry higher mutation frequencies and coherent expression shifts across ryanodine-receptor and channel components (e.g., RYR1) with positive pathway enrichment by GSEA, nominating calcium-dependent excitability as a metastasis-linked property in neuroendocrine epithelium. This aligns with broader evidence that Ca2+ circuit remodeling supports invasion and survival across cancers^36^-^38^. In parallel, WNT nodes show recurrent lesions with consistent transcriptional upregulation in metastasis, resonating with reports that WNT/β-catenin contributes to neuroendocrine tumor growth and invasiveness and is variably active in pNENs^39^, ^40^. Finally, although canonical KRAS mutations are uncommon in pNETs relative to pancreatic ductal adenocarcinoma (PDAC), our data indicate modulation of the Hallmark KRAS-signaling-DN program together with PI3K-AKT enrichment. This is consistent with the long-standing view from genomics that pNETs frequently perturb MEN1/DAXX/ATRX and mTOR-PI3K pathways rather than RAS itself^7^. Clinically, this convergence provides a mechanistic rationale for the efficacy of pathway-directed agents everolimus and sunitinib in combination with WNT or Calcium-singaling-targeting agents in metastatic, MEN1-wild type disease^14^, ^15^. Together, these findings argue that metastatic competence in low-grade pNETs reflects quantitative reinforcement of a shared circuitry rather than wholesale pathway rewiring.

Our findings identify ZNF273 and ZNF184 as a previously unappreciated Krüppel-associated box (KRAB)-zinc finger axis linked to metastatic competence and poor outcome in pNETs, extending prior genomic studies that emphasized only MEN1, DAXX, ATRX and mTOR pathway alterations^7^, ^9^, ^41^, ^42^. ZNF273 is mutated in more than half of metastatic versus fewer than one fifth of non-metastatic pNETs with metastatic cases exhibiting an accumulation of mutations in KRAB_A box domain. These patterns align with pan-cancer observations that mutations targeting structurally critical domains can rewire transcriptional programs that promote progression^43^. Growing evidence indicates that KRAB-ZNFs function as context-dependent oncogenic regulators^44^, ^45^. Our results indicate that KRAB-ZNFs may stratify metastatic risk within the otherwise genetically quiet pNET landscape and warrant functional investigation as contributors rather than incidental passengers in metastatic progression.

In this study, we used complementary pathway-level and gene-centric perturbation analyses to nominate actionable and repurposable drugs and, equally important, to flag agents predicted to be non-beneficial in metastatic disease. These results extend prior genomic characterizations that emphasized MEN1/DAXX/ATRX and PI3K-mTOR lesions in pNETs by mapping metastasis-linked transcriptional programs onto pharmacologic space rather than solely onto mutations^7^, ^9^, ^18^. Upstream-regulator analysis with iPathwayGuide^25^ revealed a list of genes that could prevent metastatic potential of pNETs as well as agents less likely to help. Moreover, Gene2Drug queries on metastasis-associated genes e.g. ZNF273 and RYR1 ranked drugs that could reverse target-pathway transcriptional activity^26^. While ZNF273 is novel in pNETs, RYR1 dysregulation is linked to tumor progression and drug repurposing in other solid tumors supporting its functional relevance^27^, ^28^. Our drug-prediction framework aligns with emerging clinical sequencing data in metastatic low-grade pNETs and offers testable hypotheses to explain therapeutic sensitivity, resistance, and agents unlikely to benefit this subset^46^. These data argue that metastasis in MEN1-wild type low-grade pNETs is accompanied by coherent, drug-modifiable transcriptional circuits^9^, ^18^.

The *in vitro* validation substantially strengthens the central argument of this study by moving ZNF273 and RYR1 from correlative candidates to functional dependencies. Both genes are overexpressed in BON1 and QGP1 cells relative to islets, and their silencing suppresses short-term viability, clonogenic outgrowth, spheroid fitness, and migration (**Figure 6**). These results identify a MEN1-wild-type metastatic program with actionable effector nodes in a KRAB-ZNF/calcium axis rather than in canonical driver genes alone^7^, ^9^, ^18^. The functional impact of ZNF273 is biologically plausible in light of growing evidence that KRAB zinc-finger proteins can act as context-dependent regulators of proliferation, invasion, stress adaptation, and metastasis-associated transcriptional programs^45^, ^47^. Mechanistically, the functional impact of RYR1 modulation is in agreement with the broader literature implicating calcium-release machinery in malignant phenotypes, including proliferation, migration, survival, and metabolic adaptation^27^. Finally, our drug-response data provide an important translational bridge. All the predicted compounds inhibited pNET growth *in vitro*, with doxorubicin showing the strongest activity in 2D and QGP1 spheroid models (**Figure 6G**-**H**). Our results complement the established benefit of pathway-directed agents such as everolimus^14^ and sunitinib^15^ in advanced pNETs and also align with prior pre-clinical data demonstrated therapeutic targeting of WNT pathway^48^. The metastatic MEN1-wild-type pNETs may require combination strategies that target both established clinical pathways and the calcium/WNT/KRAB-ZNF circuitry defined here.

Our results support ZNF273 and RYR1 as functionally relevant mediators of pNET progression and potential therapeutic vulnerabilities (**Figure 7**). Their increased expression in liver-metastatic G1/G2 pNETs suggests an association with metastatic behavior. RYR1 silencing or reduction disrupted the STIM1-ORAI1 balance and reduced intracellular Ca²⁺ signaling, implicating altered calcium homeostasis as a potential mechanism underlying pNET survival and progression. In parallel, ZNF273 depletion markedly enhanced doxorubicin sensitivity, indicating that ZNF273 may contribute to therapeutic resistance. The observed modulation of ZNF273, RYR1, and β-catenin, AKT1, AKT2, mTOR by the predicted compounds further supports target engagement and crosstalk among RYR1-dependent Ca²⁺ release, WNT/β-catenin, and PI3K-AKT-mTOR signaling (**Figure 7F** and **Supplementary Figure 15**). Importantly, fasudil and harmol inhibited BON1- and QGP1-derived xenograft growth without substantial body-weight loss, providing preclinical evidence that pharmacologic targeting of these pathways may represent a tolerable therapeutic strategy for pNETs.

This study has several limitations. The retrospective, single-institution design may introduce selection bias, while the small number of metastatic cases reduces statistical power for subgroup analyses. Functional-status and longitudinal clinical annotations were incomplete, and the limited *in vivo* functional validation and absence of prospective biomarker assessment preclude definitive conclusions. The prognostic and biomarker value of the 29-gene signature requires validation in larger, independent pNET cohorts with integrated molecular and clinical outcome data. Such studies are needed to establish its reproducibility and utility for metastatic-risk stratification. Our identified genes such as ZNF273 and RYR1 showed limited alteration compared to earlier studies that might be due to the integration of genomic and transcriptomic findings rather than mutation recurrence alone. Additionally, population ancestry could contribute to cohort-level differences, but ancestry information was not available for a rigorous association analysis. The present analysis differs from previous studies with respect to fresh-frozen versus FFPE tissue, whole-genome versus whole-exome sequencing, sequencing depth, availability of patient-matched normal DNA etc. These findings should therefore be considered hypothesis-generating and validated in larger, prospectively annotated, multi-institutional cohorts with longer follow-up and complementary functional studies.

By deeply characterizing MEN1-wild-type, low-grade pNETs, we show that metastasis could be a property of pathway state rather than simple mutation burden. Metastatic tumors exhibit slightly higher mutation frequencies but stronger activation of Calcium, WNT, and KRAS/PI3K signaling, converging on a 29-gene set that links genomic alterations to transcriptomic remodeling. Convergent activation of such programs distinguishes metastatic from non-metastatic tumors and connects directly to rational therapeutic choices. This framework shifts emphasis from single-gene events toward network activity and provides a blueprint for biomarker-guided combination trials in a clinically important subset of pNETs.

## Supplementary Material

Supplementary methods and figures.

## Figures and Tables

**Figure 1:**
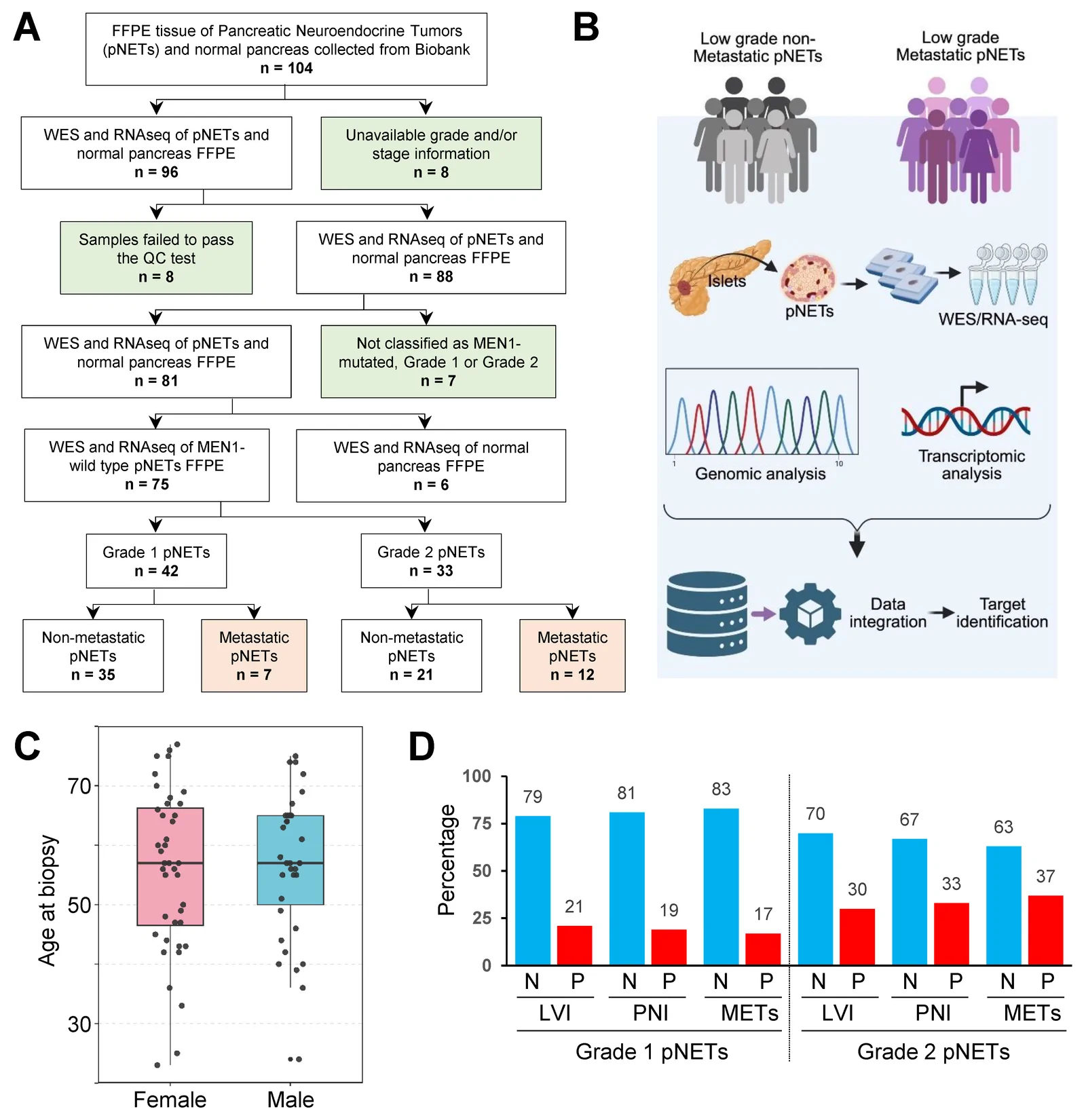
** Overview of patient inclusion and subclassification of FFPE tissues. A.** Flowchart of pNET-patient FFPE inclusions and exclusions. From low grade pNETs patient cohort, FFPE biopsy tissues from 104 distinct patients were selected. Total pool of samples was subject to whole exome sequencing (WES) and RNA sequencing (RNA-seq). The filtering criteria that led to sample exclusion are highlighted in light green. The final set of selected samples is depicted in white boxes. Metastatic samples are highlighted in light pink. **B.** Subclassification of pNET patients and samples processing schema. The 75 primary pNETs were subclassified into two major categories based on their metastatic outcome. **C.** Age distribution stratified by gender of the pNET cohort. The variable's distribution and median were visualized with data points clustered on the boxplot. **D.** Percentage of lympho-vascular invasion (LVI), peri-neural invasion (PNI) and metastasis (METs) in Grade 1 and Grade 2 pNET patients. Exact percentage shown on top of each bar. N, negative (blue); P, positive (red). Credit: **Figure 1B** was created with BioRender (https://biorender.com/).

**Figure 2:**
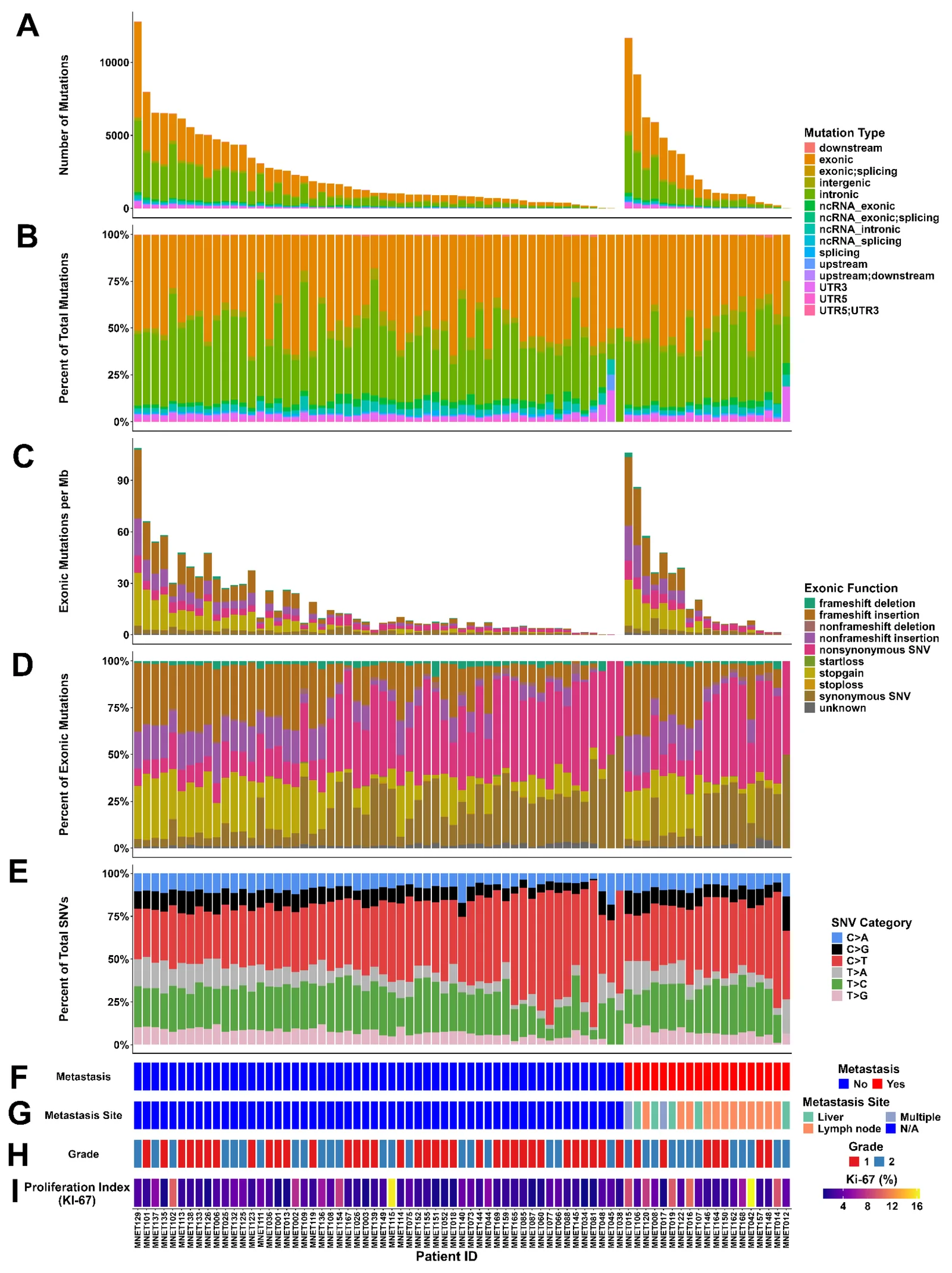
** Landscape of large-scale genomic alterations detected in pNETs, ordered by metastatic potential and differentiation grade. A.** Overview of genomic characteristics of pNETs, tumor mutational burden (TMB) ordered by metastatic potential and differentiation grade. For each pNET (n = 75), a number of genomic mutations per megabase (Mb) is shown. B. Percentage of mutations for all mutation types in all pNET samples. **C.** Overview of exonic TMB ordered by metastatic potential and differentiation grade. For each pNET (n = 75), a number of exonic Mb is shown. **D.** Percentage of exonic mutations for all mutation types in all pNET samples. **E.** Relative frequency (percentage) of pyrimidine point mutation as the total single nucleotide variants (SNVs) with six categories for all pNET samples. **F.** Categorization of primary tumors based on the patient's metastatic status. Metastatic patient samples are depicted in red and non-metastatic patient samples are depicted in blue. **G.** Categorization of metastasis based on their sites. Liver metastasis depicted in green, lymph node metastasis depicted in orange, metastasis at multiple sites depicted in light purple and non-metastatic samples marked in blue. **H.** Differentiation grade of the pNETs; grade 1 (G1) samples are shown in red and grade 2 (G2) samples are shown in blue. **I.** Proliferation index (Ki-67) from the pathological record where scores range 0-16 are represented as a gradient between dark purple as the lowest score to yellow as the highest score.

**Figure 3:**
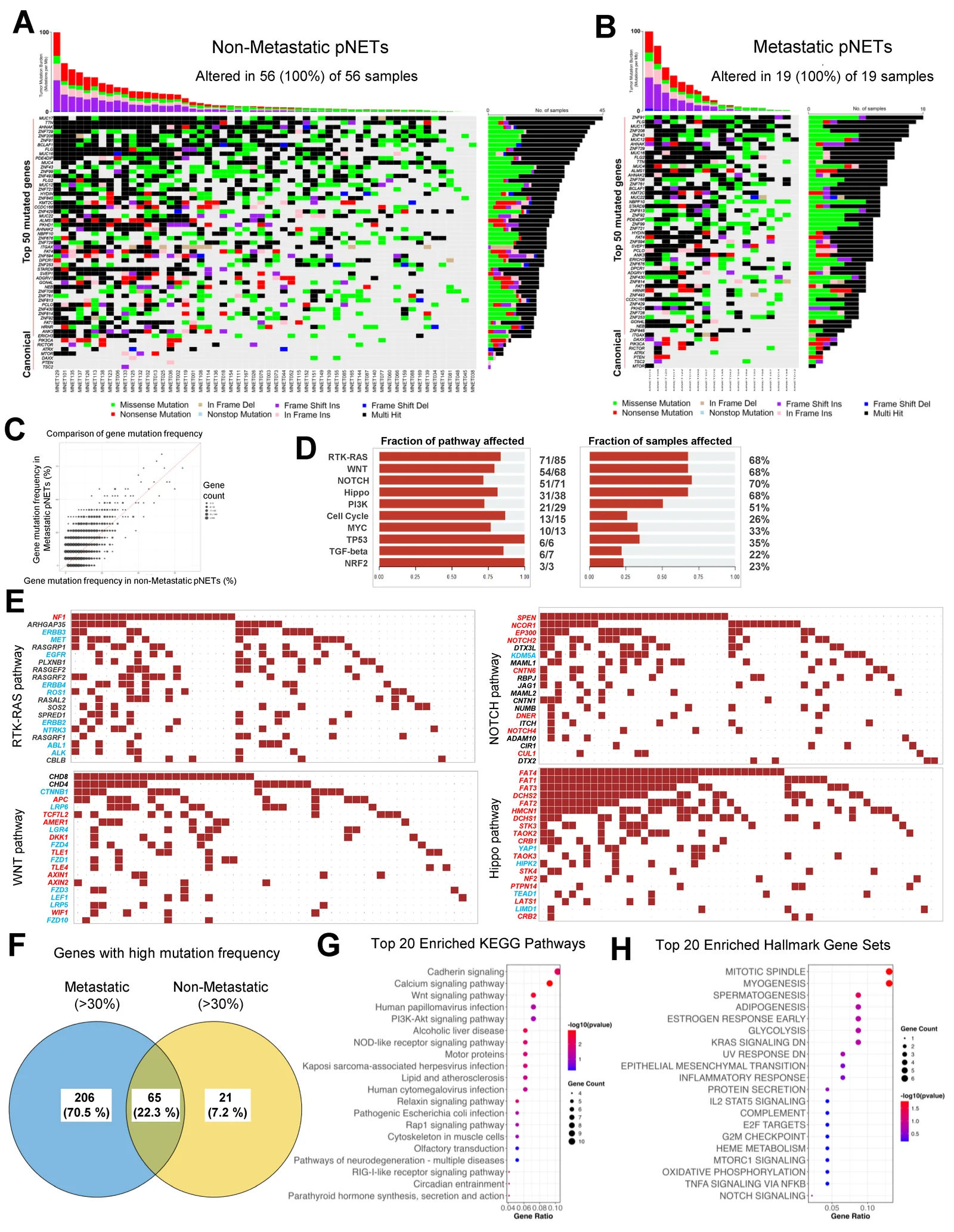
** Landscape of somatic alterations and tumor mutational burden in the pNET study cohort. A.** Oncoprint showing the top fifty recurrently mutated genes and seven canonical genes across 56 primary pNETs from the patients with non-metastatic status. **B.** Oncoprint showing the top fifty recurrently mutated genes and seven canonical genes across 19 primary pNETs from the patients with metastatic status. Each column represents an individual tumor, and each row represents a gene, ordered by decreasing mutation frequency. The stacked bar plot above the heatmap depicts tumor mutational burden (TMB; mutations per megabase) for each sample. The height of the bar reflects total TMB and the colors indicate the contribution of different mutation classes. The horizontal bar plot on the right shows the number of tumors harboring at least one mutation in each gene. Genes in the lower “Canonical” panel highlight established cancer-driver genes analyzed in this cohort. Colored boxes within the heatmap denote the mutation type observed in that gene-tumor pair. **C.** Bubble scatterplot contrasting the prevalence of recurrent gene mutations in metastatic versus non-metastatic pNETs. The mutation frequencies are expressed as the percentage of cases harboring ≥1 coding alteration. Each bubble represents one or more genes that share the same frequency pair and bubble size encodes the number of genes at that coordinate (legend, right). The red dashed diagonal marks parity between groups.** D.** Canonical oncogenic pathway alterations and functional enrichment in hypermutated pNETs. Pathways impacted by the changes in mutational landscape in pNETs compared to normal pancreas tissues. Bar plots summarize the distribution of somatic alterations across major oncogenic signaling pathways. Pathways are ordered by the decreasing number of altered genes. For each pathway, the relative fraction of alterations among all pNET samples is shown in the right panel. **E.** Gene-level mutation maps for canonical signaling pathways including RTK-RAS, NOTCH, WNT and Hippo signaling. Columns represent individual tumors and rows represent individual genes within each pathway. The red squares indicate the presence of at least one somatic mutation in that gene in the corresponding tumor. Gene names in red mark core canonical drivers, whereas those in blue highlight essential signaling or other potentially druggable components. **F.** Venn diagram depicting the overlapping mutated gene numbers between non-metastatic and metastatic patient pNET samples. Percentages representing the proportion of the combined gene set. **G.** Functional enrichment of top twenty KEGG pathways in metastatic patient pNET samples as compared to non-metastatic samples. **H.** Functional enrichment of top twenty hallmark gene sets in metastatic patient pNET samples as compared to non-metastatic samples. Each dot corresponds to a significantly enriched pathway or gene-ontology term. The x-axis denotes enrichment significance, the color scale indicates adjusted p-value (log10-transformed), and dot size reflects the number of genes from the input list contributing to each term.

**Figure 4:**
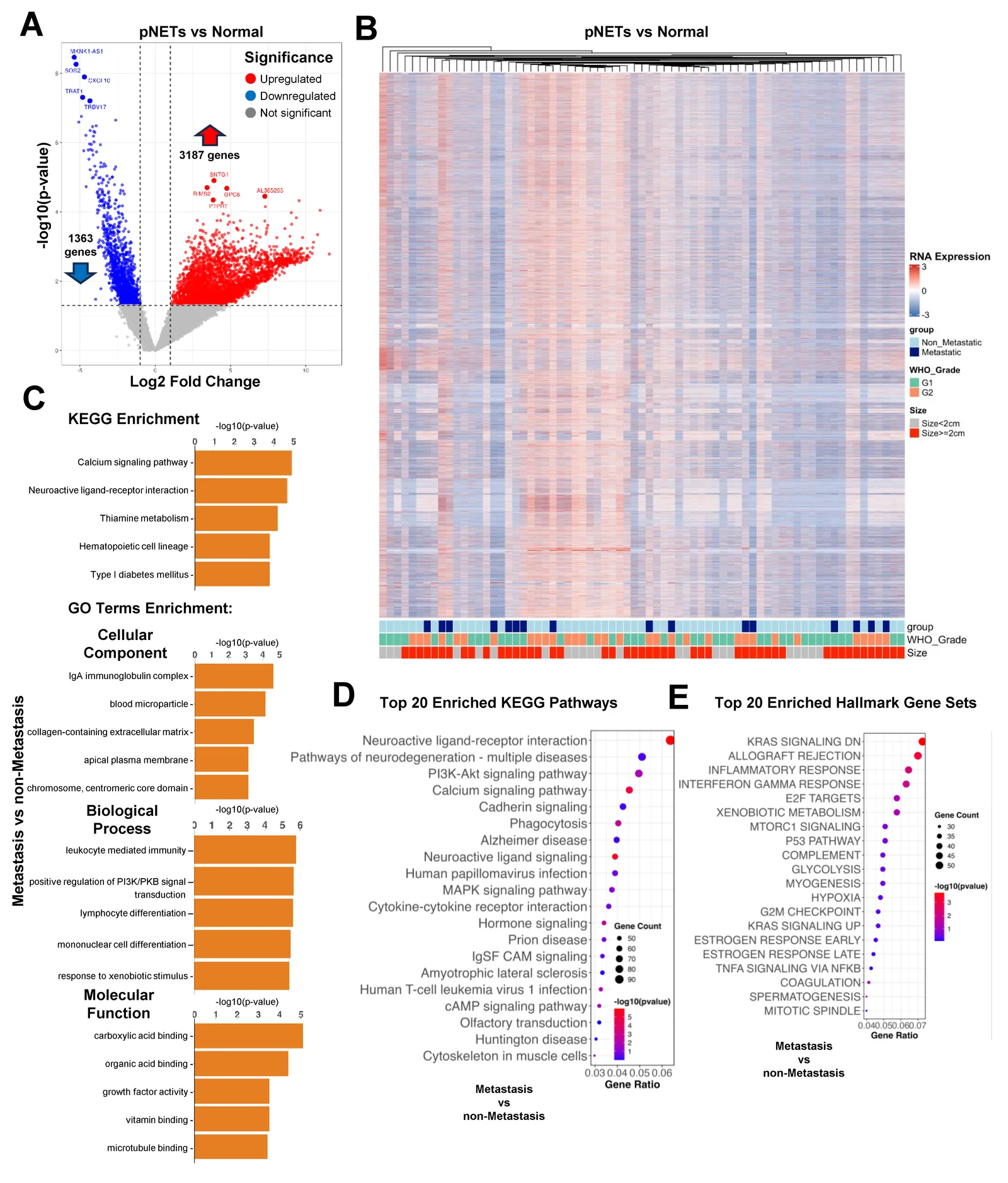
** Differential gene expressions in low-grade pNET patient samples. A.** Volcano plot of differentially expressed genes in pNET patient samples compared to normal pancreases. The x-axis shows log2 fold-changes, and the y-axis shows -log₁₀ adjusted *P* value. Red and blue points indicate significantly upregulated and downregulated genes, respectively. The top up- and- downregulated genes are labelled. **B.** Unsupervised hierarchical clustering heatmap of differentially expressed genes (DEGs). Each column represents an individual tumor and each row a gene. Relative expression is depicted in a color scale where red indicates higher expression and blue indicates lower expression. The annotation bars below the heatmap indicate clinical/pathologic features for each sample: Group, metastatic status, WHO grade (G1 and G2) and tumor size (≤2 cm vs >2 cm). **C.** Horizontal bar plots showing the functional enrichment analysis of DEG between non-metastatic and metastatic tumors. The top KEGG and Gene Ontology (GO; biological process, cellular component and molecular function) enrichments are ranked by significance.** D.** DEG associated functional enrichment of top twenty KEGG pathways in metastatic patient pNET samples as compared to non-metastatic samples.** E.** DEG-associated functional enrichment of top twenty hallmark gene sets in metastatic patient pNET samples as compared to non-metastatic samples. Each dot corresponds to a significantly enriched pathway or GO term. The x-axis denotes enrichment significance, the color scale indicates adjusted p-value (log10-transformed), and dot size reflects the number of genes from the input list contributing to each term.

**Figure 5:**
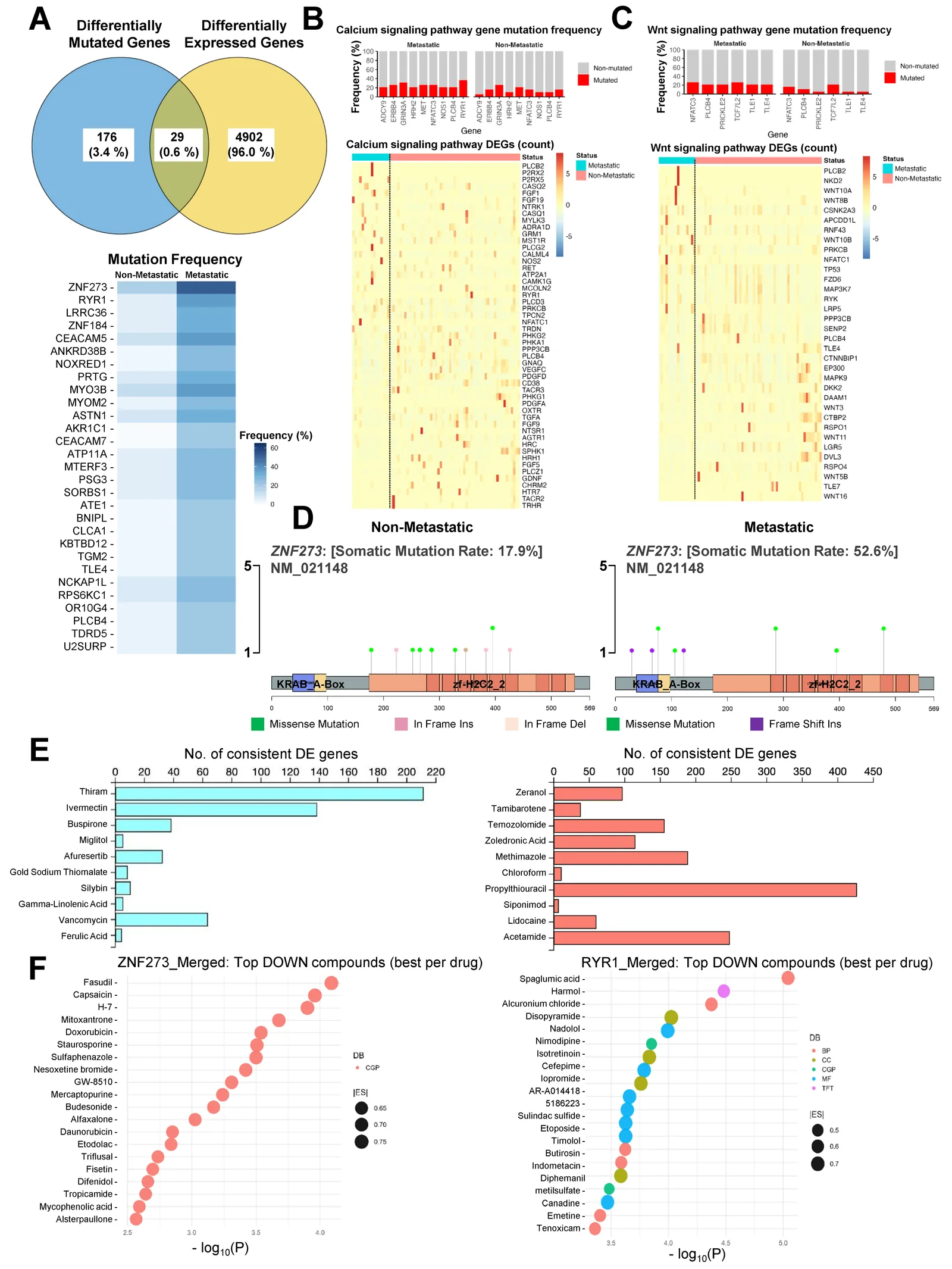
** Integration of mutational alterations and transcriptional changes derived from the same tumors highlights key genes and pathways in low-grade pNETs. A.** Venn diagram showing the overlap between significantly mutated genes and differentially expressed genes (DEGs) in low-grade pNETs. The number of genes is indicated in each segment. Mutational frequency of overlapping genes in non-metastatic and metastatic pNET samples are shown in the lower panel. **B-C.** Key pathways associated with overlapping genes. Upper panel indicates gene mutation frequencies both in non-metastatic (right) and metastatic (left) pNET samples. Lower heatmap indicates the count of DEGs in non-metastatic (pink) and metastatic (blue) pNET samples. Columns represent samples and rows represent genes. **B.** Mutation frequency and differential expression in calcium signaling pathway genes in metastatic and non-metastatic pNETs. **C.** Mutation frequency and differential expression in WNT signaling pathway genes in metastatic and non-metastatic pNETs. **D.** Lollipop plot of ZNF273 (NM_021148) somatic mutations in non-metastatic primary pNETs (n = 56) and metastatic primary pNETs (n = 19). The linear schematic depicts the ZNF273 protein with the N-terminal KRAB_A-Box domain and C-terminal zinc-finger region (zf-H2C2_2). Each lollipop represents a somatic variant identified by whole-exome sequencing, plotted at its codon position. **E.** Clinically actionable genetic alterations observed in low-grade pNETs with metastatic status. iPathwayGuide analysis (Advaita Corp. 2025) of gene expression in metastatic pNETs compared to non-metastatic pNETs predicted drugs that could have actionable changes in low-grade pNETs that have metastasized in the patients. The prediction of upstream drugs is based on the enrichment of differentially expressed genes (DEGs) from the experiment and a network of interactions from the Advaita Knowledge Base (AKB v18.1). Bar diagram indicates number of consistent DEGs (left panel). iPathwayGuide analysis of gene expression in metastatic pNETs compared to non-metastatic pNETs predicted drugs that may not have actionable changes in low-grade pNETs have metastasized in the patients. Bar diagram indicates number of consistent DEGs (right panel).** F.** The Gene2Drug software predicted multiple drugs against topmost overlapped genes (after integration of genetic alteration and gene expression data) observed in the study having high tumor mutational burden in metastatic pNETs. DOWN indicates inhibitory effect. Gene2Drug-predicted drugs against ZNF273 alterations (left panel). Gene2Drug-predicted drugs against RYR1 alterations (right panel). BP, biological pathway; CC, cellular component; CGP, cancer-related drug perturbation data; DB, database; ES, enrichment score; MF, molecular function; TFT, Transcription-factor-target sets.

**Figure 6:**
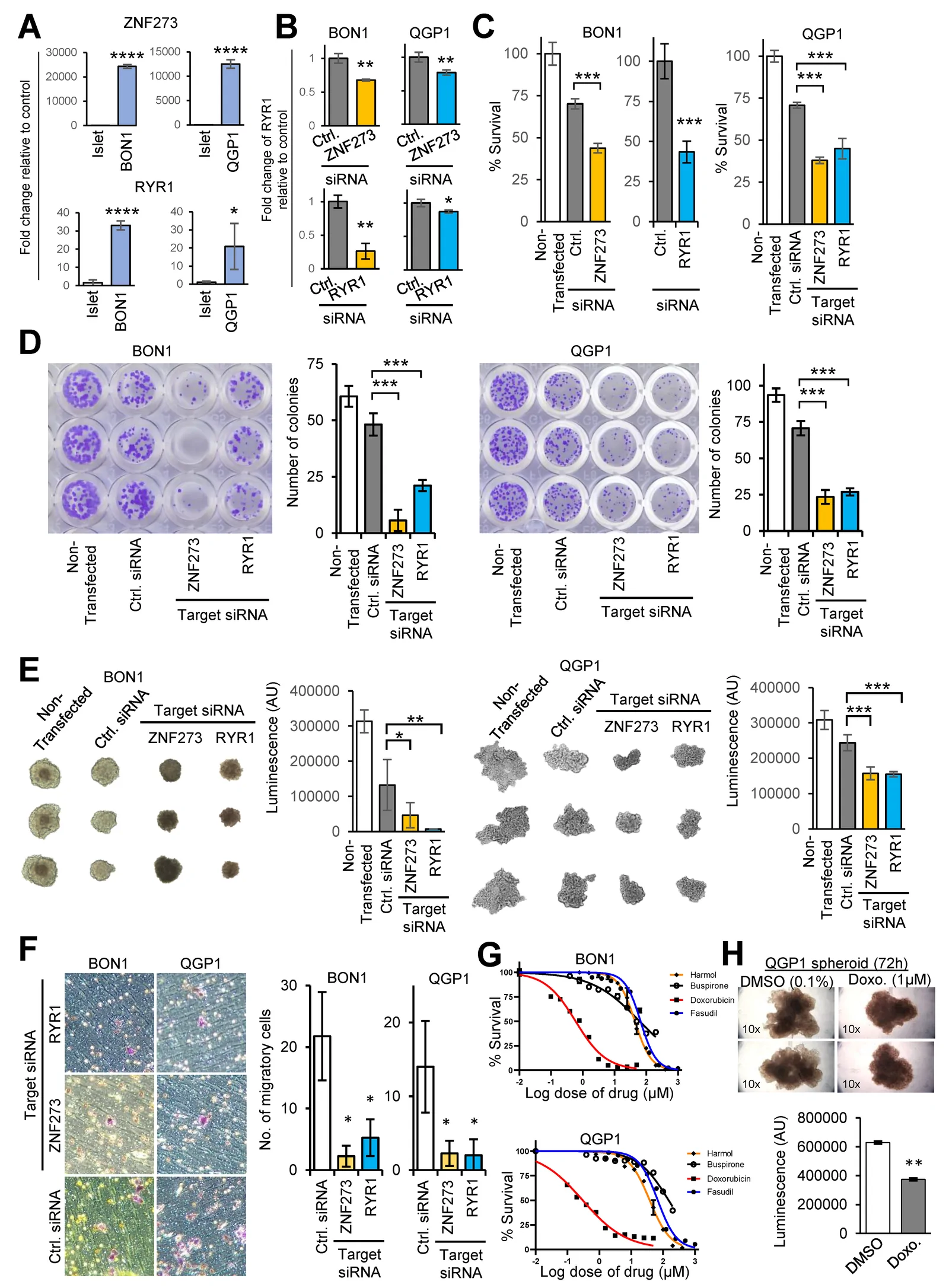
** Validation of ZNF273 and RYR1 expressions in pNET cell lines and their association with growth, clonogenicity, spheroid viability, migration, and demonstration of actionable vulnerabilities using predicted drugs. A.** RT-qPCR showing baseline expression of ZNF273 and RYR1 in BON1 and QGP1 cells relative to human islet (ABC-TC4286) control. **B.** RT-qPCR validation of siRNA-mediated knockdown of ZNF273 or RYR1 in BON1 and QGP1 cells. **C.** Cell viability measured by MTT assay 72 hours after transfection with control or target siRNAs (ZNF273 or RYR1), shown as percent survival relative to the indicated control (RYR1 silencing in BON1 cells done for 5 days). **D.** Representative crystal-violet clonogenic assays and quantification of colony number following ZNF273 or RYR1 silencing for 7-10 days compared to non-transfected and control siRNA treatment. **E.** Representative 3D spheroids and luminescence-based quantification using CellTiter-Glo assay (Promega Co.; AU, arbitrary unit) of spheroid growth/viability after ZNF273 or RYR1 knockdown (BON1 and QGP1, conditions as labeled). **F.** Representative Boyden chamber migration images and quantification of migrated cells 48 hours after siRNA transfection (control siRNA vs ZNF273 or RYR1 siRNA) in BON1 and QGP1. **G.** Dose-response viability curves for BON1 and QGP1 treated with predicted drugs including harmol, buspirone, doxorubicin, fasudil, and spaglumic acid across micromolar concentration ranges (percent survival vs log drug dose, as plotted). **H.** Representative images (10x) of QGP1 spheroids treated for 72 hours with vehicle (DMSO, 0.1%) or doxorubicin (1 µM) and luminescence-based quantification using CellTiter-Glo assay (Promega Co.; AU, arbitrary units). Each experiment was performed at least in triplicate. Data are presented as mean with standard deviation as shown. Statistical significance was assessed using an unpaired, two-tailed Student's t-test. Statistical significance is indicated in the plots with asterisks (*, *P* <0.05; **, *P* <0.01; ***, *P* <0.001 and ****, *P* <0.0001).

**Figure 7:**
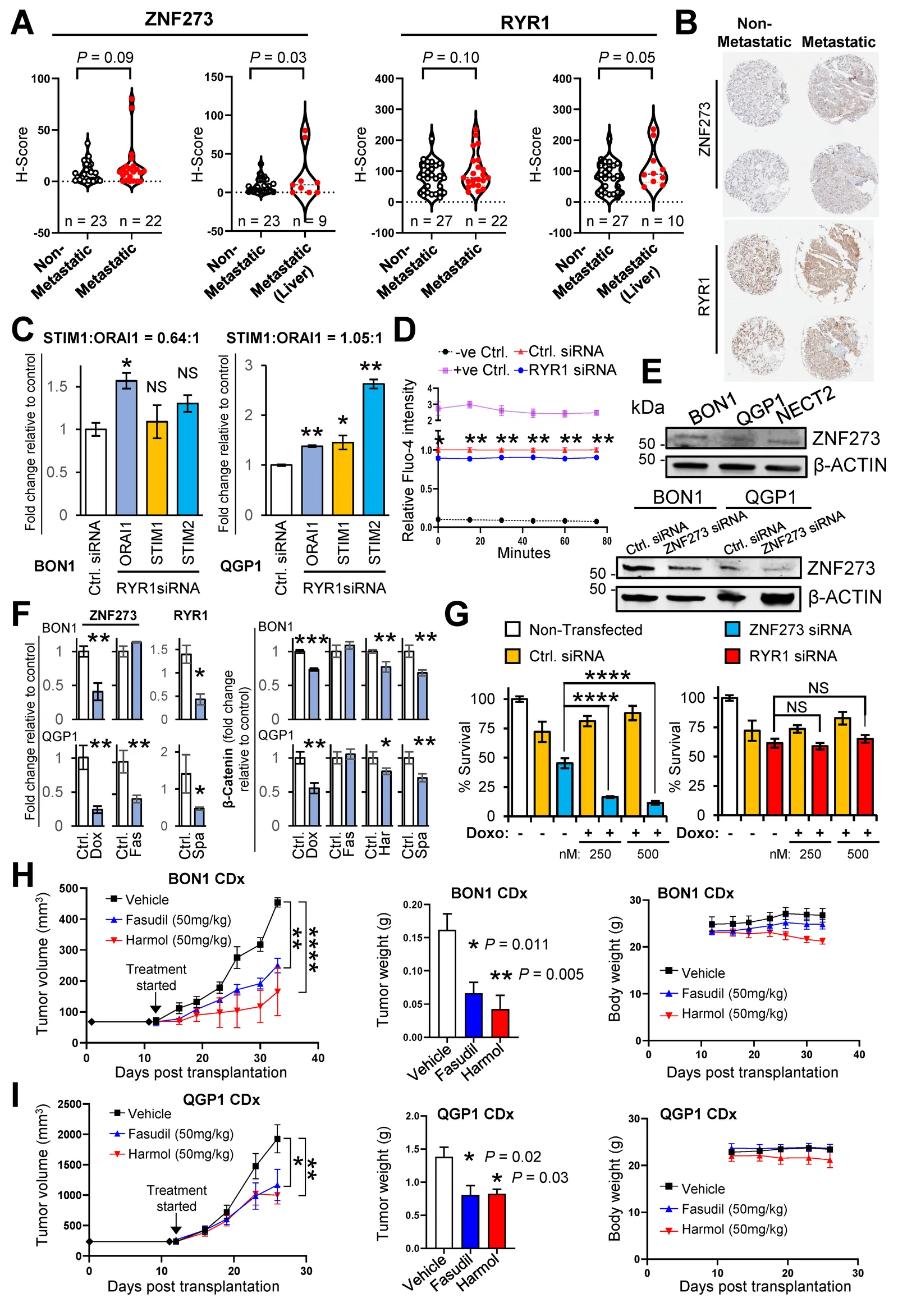
** Immunohistochemical expression of ZNF273 and RYR1 in pNET patients, functional validation and *in vivo* evaluation of predicted drugs. A.** Violin plots showing immunohistochemical H-scores for ZNF273 and RYR1 in human pNETs from patients with non-metastatic versus metastatic disease and versus the subset with liver metastasis. ZNF273 H-scores were compared between non-metastatic and metastatic tumors (n = 23 and 22, respectively including replicates for some tumors; *P* = 0.09) and between non-metastatic tumors and tumors associated with liver metastasis (n = 23 and 9 including replicates for some tumors; *P* = 0.03). RYR1 H-scores were compared between non-metastatic and metastatic tumors (n = 27 and 22 including replicates for some tumors; *P* = 0.10) and between non-metastatic tumors and tumors associated with liver metastasis (n = 27 and 10 including replicates for some tumors; *P* = 0.05). **B.** Representative immunohistochemical staining of ZNF273 and RYR1 in tissue cores from non-metastatic and metastatic pNETs. **C.** RT-qPCR analysis of ORAI1, STIM1, and STIM2 expression following RYR1 silencing in BON1 and QGP1 cells, normalized to control-siRNA condition. The calculated STIM1:ORAI1 expression ratios were 0.64:1 in BON1 cells and 1.05:1 in QGP1 cells. **D.** Time-resolved Fluo-4 fluorescence in QGP1 cells transfected with control or RYR1 siRNA, demonstrating reduced intracellular Ca²⁺-dependent fluorescence following RYR1 silencing. Assay-positive and assay-negative controls are shown. **E.** Immunoblot analysis of basal ZNF273 protein expression in BON1, QGP1 and NECT2 neuroendocrine carcinoma cells. The lower immunoblot confirms ZNF273 depletion following ZNF273-siRNA transfection in BON1 and QGP1 cells. **F.** RT-qPCR analysis of ZNF273 expression following treatment with doxorubicin (Dox) or fasudil (Fas), RYR1 expression following spaglumic acid (Spa) treatment, and β-catenin expression following treatment with Dox, Fas, harmol (Har), or Spa in BON1 and QGP1 cells. Expression values are presented relative to the corresponding vehicle-treated controls. **G.** Cell survival following ZNF273 or RYR1 silencing, alone or in combination with 250 or 500 nM Dox. Non-transfected and control-siRNA cells served as controls. ZNF273 silencing significantly reduced survival relative to control-siRNA cells at both Dox concentrations, whereas the corresponding differences following RYR1 silencing were not significant. **H.** Tumor volume, endpoint tumor weight and body weight of mice bearing BON1 cell-derived xenografts treated with vehicle, Fas, or Har. Fas and Har reduced tumor weight relative to vehicle treatment (*P* = 0.011 and *P* = 0.005, respectively). **I.** Tumor volume, endpoint tumor weight and body weight of mice bearing QGP1 cell-derived xenografts treated with vehicle, Fas, or Har. Fas and Har reduced tumor weight relative to vehicle treatment (*P* = 0.02 and *P* = 0.03, respectively). All *in vitro* experiments were performed at least in triplicate. Bars show mean values, and error bars represent SD. Statistical significance was assessed using an unpaired, two-tailed Student's t-test. Statistical significance is denoted as *P < 0.05, **P < 0.01, ***P < 0.001, and ****P < 0.0001; NS, not significant.

**Table 1:** Clinical and demographic characteristics of G1/G2 grade pNET patients.

Parameter	Sex	Frequency	Percentage (%)
Sex	Male	34	45.33
	Female	41	54.67
	Total	75	100
Median age (range)	Male	57 (51)	-
	Female	57 (58)	-
	Total	57 (58)	-
Average age	Male	57.11	-
	Female	54.76	-
	Total	55.83	-
Tumor grade 1	Male	19	45.24
	Female	23	54.76
	Total	42	100
Tumor grade 2	Male	15	45.45
	Female	18	54.54
	Total	33	100
T-stage	Male		
	Stage 1	8	-
	Stage 2	16	-
	Stage 3	9	-
	Stage 4	1	-
	Female		
	Stage 1	14	-
	Stage 2	15	-
	Stage 3	9	-
	Stage 4	2	-
	Undetermined	1	-
N-stage	Male		
	Stage 0	21	-
	Stage 1	11	-
	pNx	2	-
	Female		
	Stage 0	28	-
	Stage 1	10	-
	pNx	2	-
	N/A	1	-
LN-status	Male		
	Positive	11	-
	Negative	21	-
	N/A	2	-
	Female		
	Positive	11	-
	Negative	27	-
	N/A	3	-
Metastasis	Male		
	Yes	10	-
	No	22	-
	N/A	1	-
	Undetermined	1	-
	Female		
	Yes	9	-
	No	31	-
	N/A	1	-

Abbreviations: T-stage, tumor stage; N-stage, nodal stage; LN, lymph node; N/A, not applicable; pNx, undetermined nodal status.

## Data Availability

The genetic variant data from WES of the pNET tissue samples are available from EBI's (European Bioinformatics Institute) BioStudies repository under accession number S-BSST1766 (URL: https://www.ebi.ac.uk/biostudies/studies/S-BSST1766?key=0a84424e-187f-4c36-bc01-2d59ca9669b1). The RNA-Seq data from the FFPE and fresh pNET and adjacent normal tissues are available from EBI's ArrayExpress repository under accession number E-MTAB-14709 (URL: https://www.ebi.ac.uk/biostudies/arrayexpress/studies/E-MTAB-14709?key=3a110a96-fca4-438e-822f-5afe8aab97ca). All data generated or analyzed during this study will be made available along with code information on reasonable request for academic use.
