# Supplementary material for: Genomic and Transcriptomic Landscapes of MEN1-Wild-Type Low-Grade Metastatic Pancreatic NETs Uncover Key Oncogenic Drivers and Targetable Pathways
Source: Int J Biol Sci. 2026 Sep 3;22(14):7908–27. doi: 10.7150/ijbs.134269 (PMC13618231; doi:10.7150/ijbs.134269)
Supplement: Supplementary file 1 [file ijbsv22p7908s1.pdf]

# Supplementary Information:

## Genomic and Transcriptomic Landscapes of MEN1-Wild-Type Low-Grade Metastatic Pancreatic NETs Uncover Key Oncogenic Drivers and Targetable Pathways

Md Hafiz Uddin[1#], Zaid Mahdi[2#], Irfana Muqbil[3#], Brendon R. Herring[4#], Bart Rose[4], Husain Y. Khan[1], Yiwei Li[1], Amro Aboukameel[1], Sahar F. Bannoura[1], Hugo Jimenez[1], Allan M. Johansen[5], Mohammad Najeeb Al-Hallak[1], Ibrahim Azar[1,6], Amr Mohamed[7], Tarik Hadid[1], Nitin Vaishampayan[1], Yang Shi[1], Yin Wan[1], Vy Ong[1], Gregory Dyson[1], Rafic Beydoun[8], Miguel Tobon[1], Eliza W. Beal[1], Herbert Chen[4], Anthony F. Shields[1], Philip A. Philip[1,9], Jennifer Beebe-Dimmer[1], Ramzi M. Mohammad[1], Boris C. Pasche[1], Bassel F. El-Rayes[4]*, Asfar S. Azmi[1]*

*Corresponding author
#authors contributed equally

**Affiliations:** [1]Karmanos Cancer Institute, Detroit, MI, [2]Emory Winship Cancer Institute, Atlanta, GA, [3]Lawrence Technological University, Southfield, MI, [4]O'Neal Comprehensive Cancer Center, University of Alabama at Birmingham, Birmingham, AL, [5]Department of Cancer Biology, Wake Forest University, Winston-Salem, NC, [6]Trinity Health Oakland, Pontiac , MI, [7]UH Seidman Cancer Center, University Hospitals, Case Western Reserve University, Cleveland, OH, [8]Department of Pathology, Wayne State University School of Medicine, Wayne State University, Detroit, MI [9]Henry Ford Health System, Detroit, MI.

***Address of Correspondence:**
Bassel F. El-Rayes, MD
Albert F. LoBuglio Endowed Chair for Translational Cancer Research
Division Director, Hematology and Oncology
Deputy Director, O'Neal Comprehensive Cancer Center
Heersink School of Medicine, UAB
Email: belrayes@uabmc.edu

Asfar S. Azmi, PhD.
Department of Oncology
Wayne State University School of Medicine
4100 John R, HWCRC 740.2
Karmanos Cancer Institute
Detroit, MI 48201
Tel: +1313 258 6148
Email: azmia@karmanos.org

## SUPPLEMENTARY METHODS

### Collection of pathological records:

Low grade pNETs were defined according to WHO 2017/2019/2022 criteria as well-differentiated G1 or G2 pNETs, with G1 defined as Ki-67 < 3% and G2 as Ki-67 3%-20%. All the tumors were staged using the AJCC 8th-edition TNM classification system. Regional lymph-node involvement was recorded as N1 and was distinguished from distant metastasis. Distant metastases were categorized as liver-only, extrahepatic-only, or combined hepatic and extrahepatic disease. Patients classified as nonmetastatic or metastatic were followed up for 24 months.

### Whole exome sequencing:

Exome capture was performed using the SureSelect Human All Exon V6 Kit (Agilent Technologies) following the vendor's protocol. Sequencing was performed using the Illumina Hiseq X Ten with 150-bp paired-end reads for a total of 40 million reads. Prior to alignment, the low-quality reads were removed. Reads were aligned to the reference genome using Burrows-Wheeler Alignment (BWA). Picard tools were utilized to identify and remove duplicate reads from the BAM files generated by BMA. The resulting BAM files were indexed and sorted by samtools.

### Mutational signature analysis:

Somatic variant calls were generated with GATK Mutect2, which assigned an accurate confidence score to each putative mutation call and evaluated new potential variants. Biological functional annotations for the identified somatic variants were performed using Annovar. Somatic copy number variants (CNVs) were detected using Facets. The R Bioconductor package, Maftools were utilized for summarizing, analyzing and visualizing. Mutation Annotation Format (MAF) files generated from GATK, which created the oncoplots, transition

and transversion plots, and lollipop mutation diagrams to visualize the somatic mutations in pNET patients.

**RNA-sequencing:**

For total RNA sequencing, RNA integrity was checked with Agilent Technologies 2100 Bioanalyzer to ensure RIN >7 cutoff value. All the samples passed the quality control except four pNET samples. Approximately 10 µg of total RNA was used to isolate poly(A) mRNA employing poly-T oligo–conjugated magnetic beads (Invitrogen). The captured poly(A)+ or poly(A)- RNA fractions were fragmented into short fragments using divalent cations under elevated temperature. The resulting RNA fragments were then reverse transcribed to generate complementary DNA (cDNA) libraries according to the manufacturer's protocol (Illumina, San Diego, CA, USA). The sequence library was prepared following Illumina's TruSeq-stranded-total-RNA-sample preparation protocol. Quality control analysis and quantification of the sequencing library were performed using Agilent Technologies 2100 Bioanalyzer High Sensitivity DNA Chip. Paired-end sequencing was performed on Illumina's NovaSeq 6000 sequencing system for a total of 40 million reads per sample. The expressions of transcripts generated from the same gene were aggregated to obtain the expression level of the gene. Differentially expressed genes were determined using the R Bioconductor package, applying the thresholds of absolute values of log2 (fold change) > 1 and a false discovery rate (FDR, using the Benjamini-Hochberg method) < 0.05 for statistical significance.

**Inventory of clinically actionable putative therapeutic targets against somatic alterations:**

Differentially expressed genes (DEGs) were derived from metastatic versus non-metastatic low-grade pNETs with gene symbols harmonized to HGNC. In iPathwayGuide, DEGs were mapped onto the Advaita Knowledge Base and analyzed via impact analysis, which combines (i) over-representation statistics and (ii) perturbation of pathway topology to model directionality across signaling cascades. In parallel, Gene2Drug queried the subset of

"overlapped" genes (significantly mutated and differentially expressed in the same tumors) plus pathway-adjacent DEGs against LINCS/Connectivity-Map derived perturbational signatures across multiple annotation spaces (cancer gene perturbations, Gene Ontology, transcription factor target sets etc). For each compound, an enrichment score was computed over rank-ordered drug-response profiles.

***In vitro* assays:**

**Cell and 3D viability assay:**

BON1 and QGP1 cells were cultured in DMEM/F12 and RPMI-1640 media respectively with 10% fetal bovine serum and 1% penicillin/streptomycin. Cell and 3D viabilities were measured using 3-(4,5 dimethylthiazol-2-yl)−2,5-diphenyltetrazolium bromide (MTT) and cell-titer-glo (Promega) assay respectively as described earlier [1]. In brief, cells were plated onto 96-well plates at a density of 4000 cells/well. After 72 hours of treatment, MTT solution added and incubated for 2 hours at 37.C. The formazan crystals were dissolved in dimethyl sulphoxide, the optical density was measured at a wavelength of 570 nm using a spectrophotometer.

**Colony formation assay:**

Cells were plated in 24-well plates at 100-200 cells per well and transfected with the indicated siRNAs for 4 days. The treatment medium was then replaced with fresh complete growth medium, and cultures were maintained for an additional 7-10 days to permit colony development. Colonies were subsequently fixed in methanol, stained with crystal violet for 15 minutes, washed thoroughly, and allowed to air-dry. The plates were then imaged for documentation and analysis of colony formation.

**Spheroid formation assay:**

BON1 and QGP1 cells were seeded into ultra-low-attachment 96-well plates at 25 cells per well, with each condition prepared in triplicate. Cells were transfected with the indicated

siRNAs the following day and maintained in supplemented three-dimensional spheroid culture medium (PromoCell, catalog no. C-28077) to support spheroid development. After 10-14 days, spheroids were photographed using an Olympus phase-contrast microscope equipped with a digital camera. Spheroid area was quantified from the acquired images using NIH ImageJ software.

**Trans-well migration assay:**

Cell migration was assessed using a Boyden chamber assay. Cells were suspended in serum-free medium and seeded into the upper chamber of inserts containing a 8 µM pore-size membrane, while complete medium containing serum was added to the lower chamber as a chemoattractant. After incubation, non-migrated cells remaining on the upper membrane surface were gently removed. Cells that had migrated to the lower surface were fixed, stained with crystal violet, imaged by light microscopy, and quantified in randomly selected fields.

**RT-qPCR analysis:**

Real-time reverse transcriptase quantitative PCR (RT-qPCR) was performed as described earlier [2].

**Western blot analysis:**

Western blot analysis was performed as described earlier [1]. The membranes were incubated with anti-ZNF273 (#PA5-69709, Thermofisher, MA, USA) and anti-beta-ACTIN (#sc-47778; Santa Cruz Biotechnology, Santa Cruz, CA, USA) was used at a dilution of 1:1000.

**Intracellular calcium flux assay:**

Cells were seeded (150,000 cells/well) into six-well plates, left to adhere overnight, and cultured RPMI-1640 supplemented with 10% FBS and 1% Penicillin-Streptomycin. Cells were treated with control siRNA or RYR1 siRNA for 3 days. On the day of experiment, Fluo-4 stain

mix was added directly to each well (Fluo-4 Calcium Imaging Kit; Molecular Probes; Catalog No. F10489). Fluo-4 stain was not added to the negative control wells. To the positive control wells, 5 µl of ionomycin was added. All plates were then placed on a rocker at room temperature for 1 min of gentle distribution of Fluo-4 stain. Plates were then placed back into the 37 °C incubator and incubated for 30 min. Afterwards, the plates are placed at room temperature (RT) for 30 min (in the dark). Cells were then washed using 1 ml of Live Cell Imaging Solution (LCIS; Molecular Probes; Catalog number A59688DJ). Two ml of fresh LCIS were added to each well. Fluorescent intensity was read using Agilent BioTek Synergy H1 plate reader.

**Immunohistochemistry:**

Immunohistochemical analysis was performed as described earlier [1]. The TMA tissues were incubated with anti-ZNF273 (#HPA077549, Millipore Sigma, USA) and anti-RYR1 (#HPA056416, Millipore Sigma, USA) antibodies and were used at dilution of 1:32 and 1:50 respectively.

**Immunofluorescence assay:**

Immunofluorescence analysis was performed as described earlier [2]. The pNET cells were incubated with anti-RYR1 (#8153; Cell Signaling Technology, Danvers, MA, USA) antibody upon silencing with RYR1 siRNA. and was used at a dilution of 1:200.

***In vivo* cell line-derived xenograft (CDx) models:**

Post adaptation in our animal housing facility, 4-5 weeks old female ICR-SCID mice (Taconic Biosciences, Rensselaer, NY) were subcutaneously implanted with BON1 or QGP1 cells. 1x106 cells suspended in 200 µL PBS were injected unilaterally into the left flank of donor mice using a BD 26Gx 5/8 1ml Sub-Q syringe. Once the tumors reached about 5-10% of the donor mice body weight, the donor mice were euthanized, tumors were harvested, and fragments were subsequently implanted into recipient mice. Seven days post-transplantation, the recipient mice were randomly divided into three groups of 5 mice each and received either vehicle, or

Fasudil (50 mg/kg QD, IP), or Harmol (50 mg/Kg QD, IP), for four weeks. In addition to double randomization, to further reduce bias, blinding was observed during tumor measurement and data analysis. Twenty-four hours of completion of drug dosing, tumors were harvested from control or treatment groups for further analysis.

**Statistical analysis:**

For WES data, TMB was computed as somatic single-nucleotide variants and short indels per callable megabase in the exome; base-substitution spectra were summarized in the six canonical SNV classes. Differential mutated genes between the pNET and normal samples were identified using the Fisher's exact test with a p-value $< 0.05$ and the difference in mutation frequencies between the two groups greater than 20%. The R Bioconductor package clusterProfiler was used for functional enrichment analysis, where the over-representation tests were used for KEGG (Kyoto Encyclopedia of Genes and Genomes) pathways and Gene Ontology (GO) categories and gene set enrichment analysis (GSEA) was performed for the Molecular Signatures Database (MSigDB) Hallmark gene set collections. Significantly enriched pathways, GOs and gene sets were identified using FDR$< 0.05$.

## SUPPLEMENTARY FIGURES

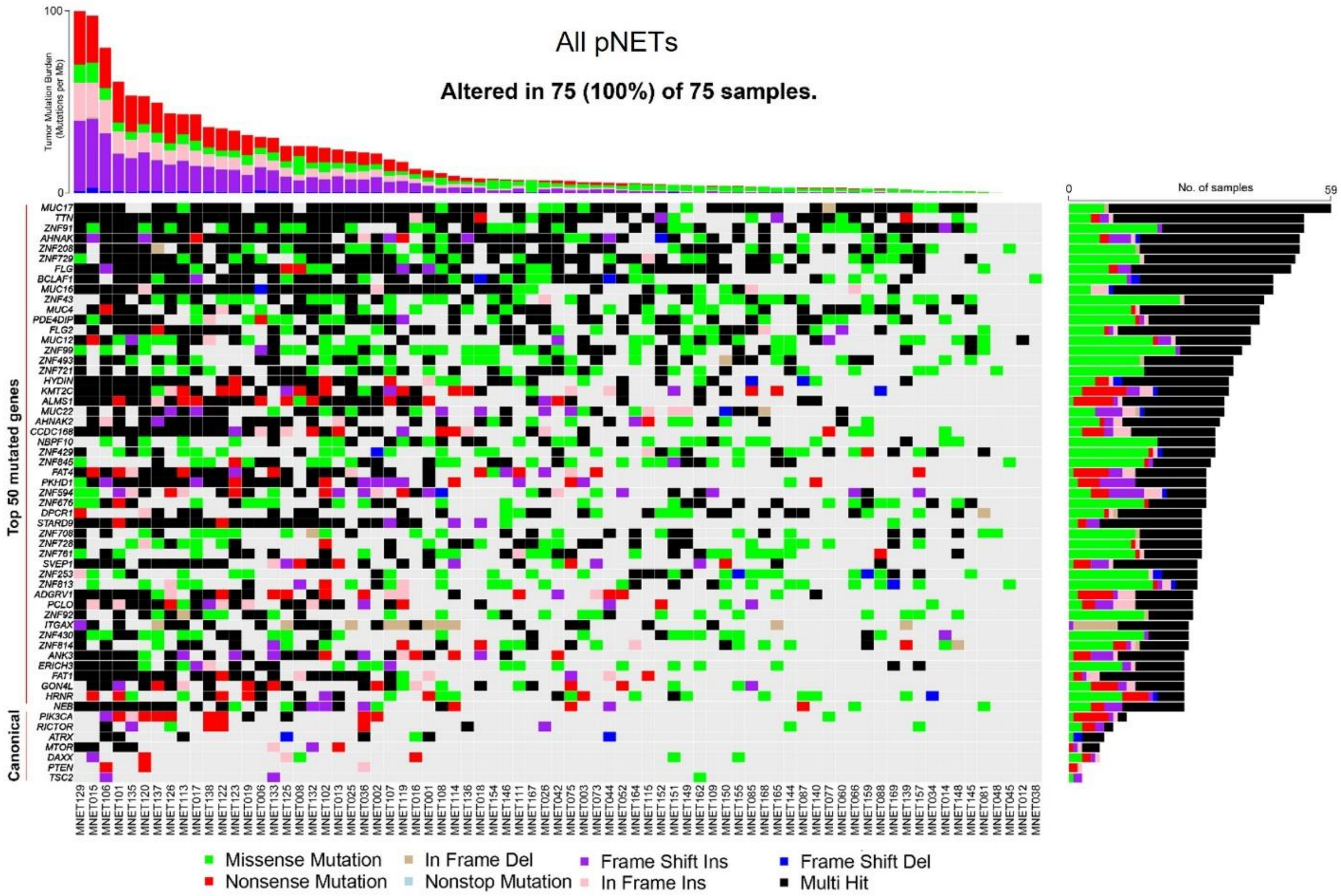


**Supplementary Figure 1. Global landscape of recurrent somatic alterations in all low-grade pNETs.** Oncoprint summarizing the top 50 recurrently mutated genes together with canonical cancer genes across all primary pNETs profiled by whole-exome sequencing (n = 75). Each column represents an individual tumor and each row a gene, ordered from top to bottom by decreasing mutation frequency. The stacked bar plot above the oncoprint depicts the tumor mutational burden (TMB) for each sample; bar height reflects overall TMB and colors indicate the contribution of different mutation classes. The horizontal bar plot to the right shows the number of tumors harboring at least one mutation in each gene. Colored boxes within the heatmap denote the mutation type identified in that gene-tumor pair: missense mutation (green), nonsense mutation (red), in-frame deletion (brown), nonstop mutation (light blue), frameshift insertion (purple), in-frame insertion (pink), frameshift deletion (blue), and multi-hit events (black). Grey cells indicate no detectable coding mutation in that gene in the corresponding tumor.

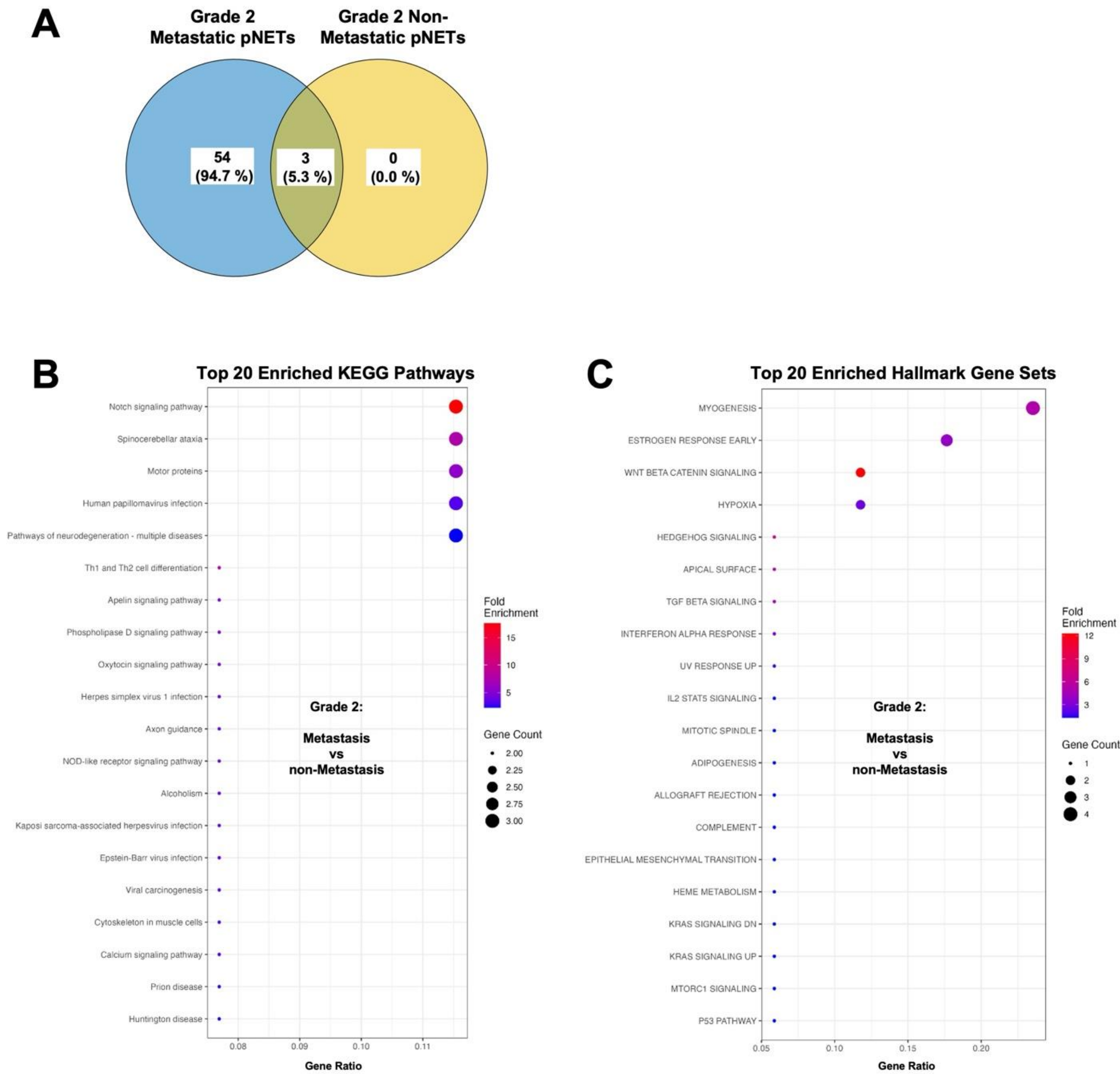


**Supplementary Figure 2. Comparison of mutational landscapes in Grade 2 metastatic versus non-metastatic pNETs. A.** Venn diagram summarizing the distribution of recurrently mutated genes in WHO Grade 2 metastatic and non-metastatic pNETs. Numbers and percentages indicate the count and proportion of genes mutated exclusively in metastatic tumors, shared between both groups, or unique to non-metastatic tumors. **B.** Dot plot showing the top 20 KEGG pathways enriched among genes mutated in Grade 2 metastatic versus non-metastatic pNETs. The x-axis depicts the gene ratio (number of mutated genes in the pathway divided by the total number of mutated genes used for enrichment). Dot size encodes the number of genes contributing to each pathway, and color represents fold enrichment, with warmer colors

indicating stronger enrichment. **C.** Dot plot of the top 20 enriched Hallmark gene sets derived from the same comparison (Grade 2 metastatic versus non-metastatic pNETs). Axes and symbol encodings are as in panel B.

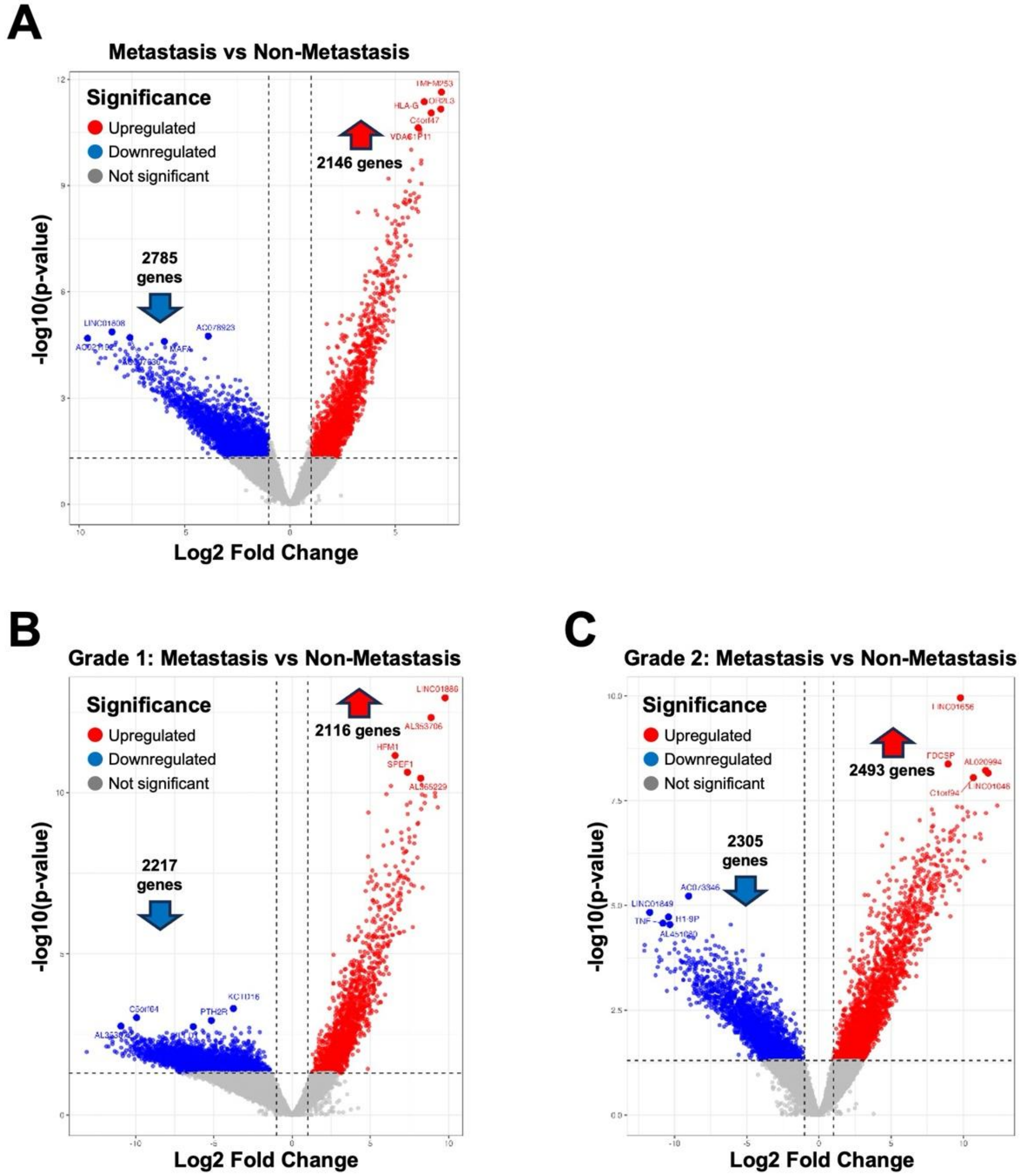


**Supplementary Figure 3. Differential gene-expression profiles associated with metastatic status in low-grade pNETs. A.** Volcano plot depicting differentially expressed genes (DEGs) between all metastatic and all non-metastatic pNETs. The x-axis shows $\log_2$ (fold change) for metastatic versus non-metastatic tumors and the y-axis shows $-\log_{10}$(*p*-value). Significantly upregulated genes in metastatic tumors are shown in red, significantly downregulated genes in blue, and non-significant genes in grey (significance threshold defined by the vertical and horizontal dashed lines). Arrows indicate the total number of significantly upregulated (2,146 genes, red arrow) and downregulated (2,785 genes, blue arrow) transcripts, with representative

top-ranked genes labeled. **B.** Volcano plot of DEGs comparing Grade 1 metastatic versus non-metastatic pNETs, displayed as in panel A. A total of 2,116 genes are significantly upregulated and 2,217 genes are significantly downregulated. **C.** Volcano plot of DEGs comparing Grade 2 metastatic versus non-metastatic pNETs. Using the same axes and color coding, 2,493 genes are significantly upregulated, and 2,305 genes are significantly downregulated in metastatic tumors.

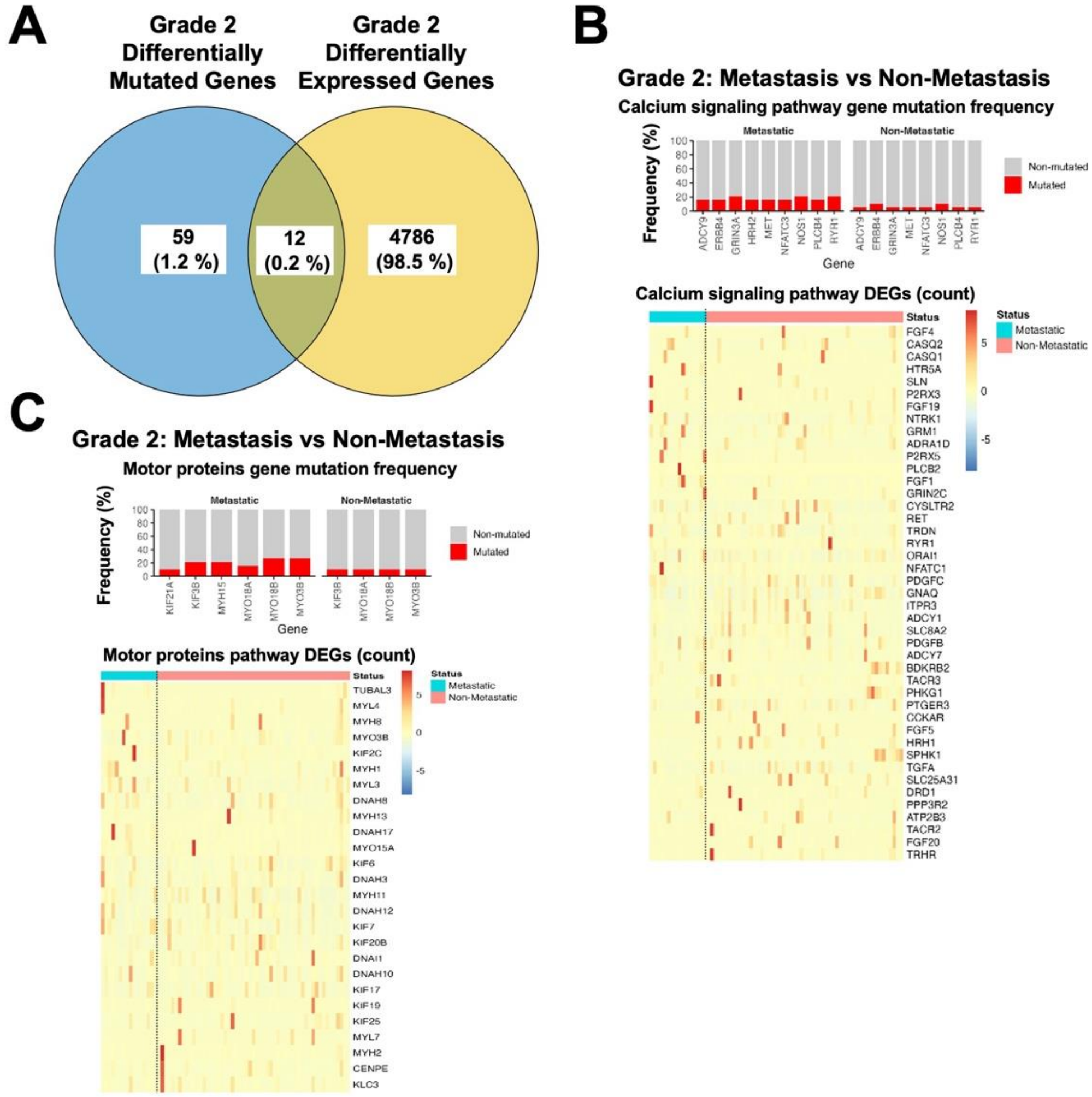


**Supplementary Figure 4. Integrated mutational-transcriptional analysis highlights calcium signaling and motor protein pathways in Grade 2 pNETs. A.** Venn diagram integrating somatic mutation and gene-expression data for all WHO Grade 2 pNETs. The blue circle denotes genes that are significantly differentially mutated between Grade 2 metastatic and non-metastatic tumors, and the yellow circle represents genes that are significantly differentially expressed (DEGs) in the same comparison. Numbers and percentages indicate the counts and proportions of genes unique to the mutational set, unique to the DEG set, or overlapping between both. **B.** Calcium signaling pathway genes identified from the overlapping set-in panel A. The upper bar plot shows the frequency of somatic mutations in each calcium pathway gene for Grade 2 metastatic and non-metastatic tumors. The lower heatmap displays the corresponding expression patterns for calcium pathway DEGs across individual tumors, with columns representing samples and rows representing genes. Expression changes are color coded with red indicating higher and

blue indicating lower expression relative to the cohort mean. **C.** Motor protein pathway genes derived from the same integrated analysis. The upper bar plot depicts mutation frequencies for motor protein encoding genes in Grade 2 metastatic and non-metastatic tumors, as in panel B. The lower heatmap shows the expression profiles of motor protein pathway DEGs in the same samples, using the same color scale.

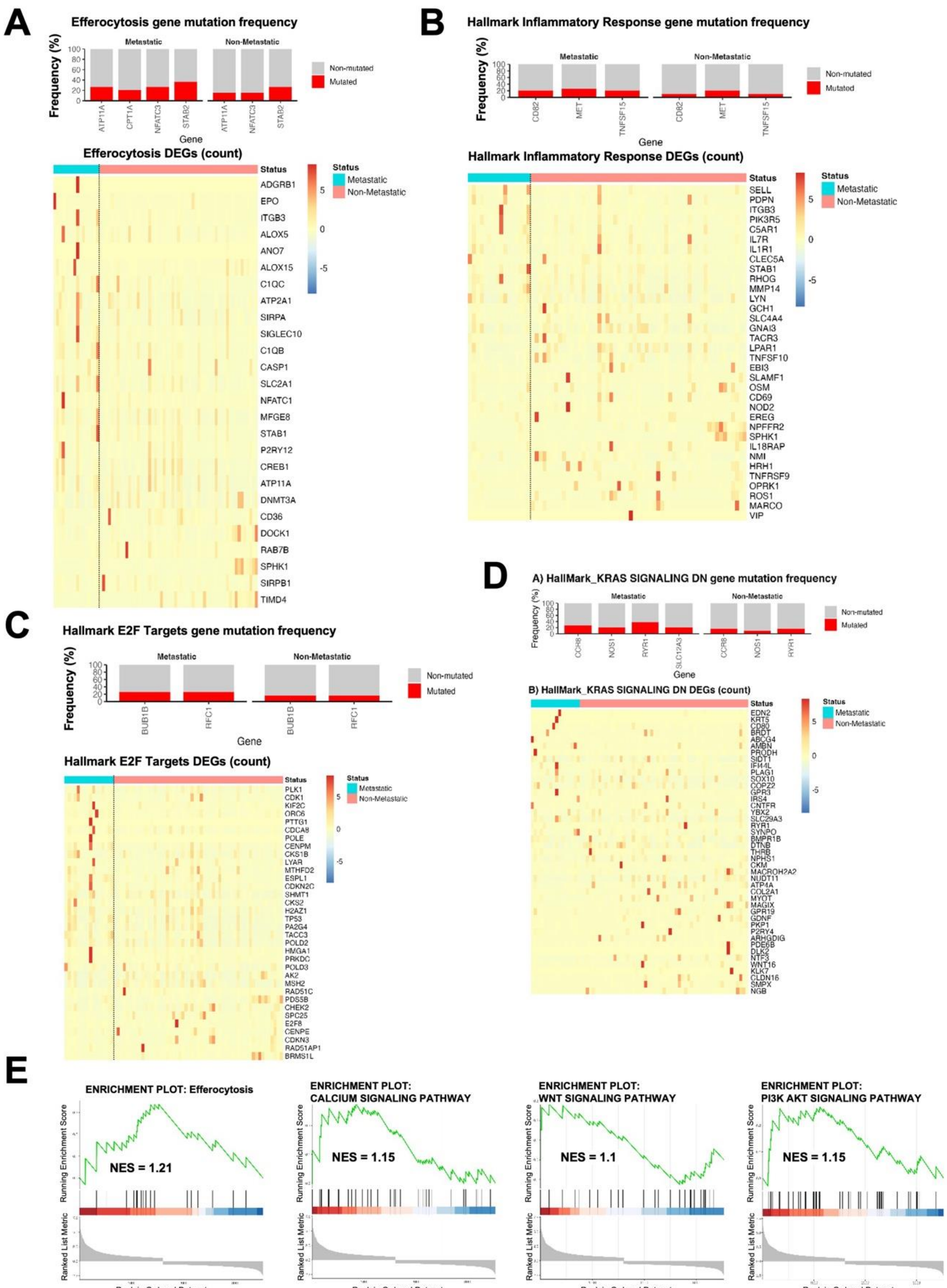


**Supplementary Figure 5. Integrative mutational and transcriptional analysis highlights efferocytosis, inflammatory response, and E2F targets programs in metastatic pNETs. A.**

Efferocytosis pathway genes identified from the overlap of differentially mutated and differentially expressed genes between metastatic and non-metastatic pNETs. The upper bar plot shows, for each gene, the proportion of metastatic (left) and non-metastatic (right) tumors carrying at least one coding mutation. The lower heatmap displays expression changes for efferocytosis-related DEGs across individual tumors, with columns representing samples and rows representing genes. Expression values are color coded, where red indicates higher and blue lower expression relative to the cohort mean. **B.** Hallmark "Inflammatory Response" gene set derived from the same integrated analysis. The upper bar plot shows mutation frequencies for selected inflammatory-response genes in metastatic and non-metastatic tumors, as in panel A. The lower heatmap depicts expression profiles of inflammatory-response DEGs across the cohort, using the same color scale. **C.** Hallmark "E2F Targets" gene set analyzed analogously. Mutation frequencies for representative E2F target genes are shown in the upper bar plot, and the lower heatmap displays expression of E2F target DEGs in metastatic and non-metastatic tumors. **D.** Hallmark KRAS-signaling-DN gene set and association with overlapped genes. Upper panel indicates gene mutation frequencies both in non-metastatic (right) and metastatic (left) pNET samples. Lower heatmap indicates the count of DEGs in non-metastatic (pink) and metastatic (blue) pNET samples. Columns represent samples and rows represent genes. **E.** Gene set enrichment analysis (GSEA) plot for the efferocytosis pathway, calcium signaling, WNT signaling and PI3K-AKT signaling pathways, in metastatic versus non-metastatic pNETs. The running enrichment score (green line) is plotted as a function of the ranked gene list, with the vertical bars indicating positions of respective genes and the normalized enrichment score (NES) summarizing pathway-level upregulation in metastatic tumors.

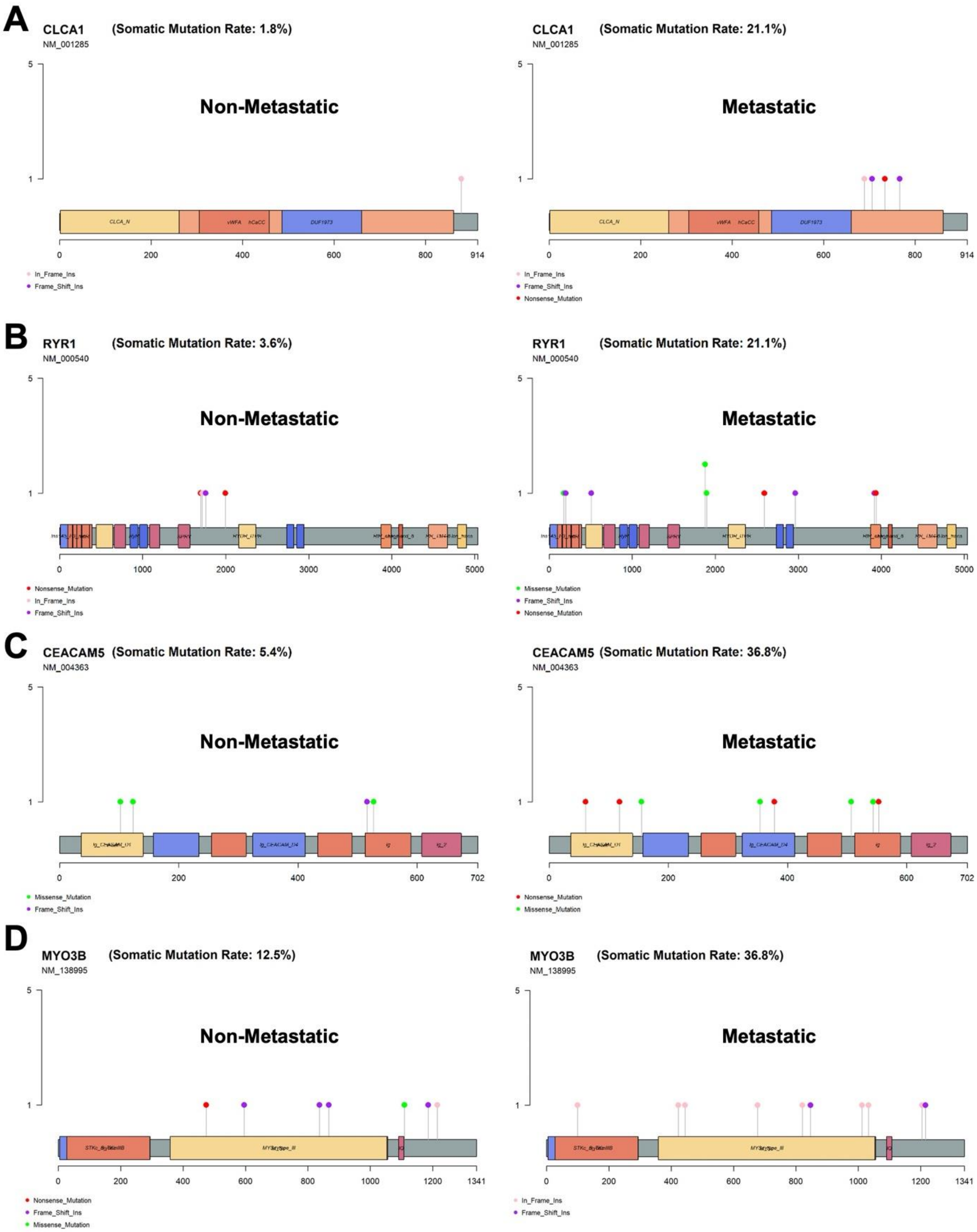


**Supplementary Figure 6. Enrichment and qualitative shift of CLCA1, RYR1, and CEACAM5 coding alterations in metastatic MEN1-wild-type low-grade pNETs.** Lollipop

plots depict somatic mutations in selected genes in primary human pNETs profiled by whole-exome sequencing (non-metastatic, n = 56; metastatic, n = 19). For each gene, the left plot shows non-metastatic tumors, and the right plot shows metastatic tumors. The horizontal schematic represents the annotated protein with major domains indicated and amino-acid positions shown on the x-axis; each lollipop marks the position of a distinct variant. Colors denote mutation class as indicated in the below. Somatic mutation rates for each gene and group are reported in the panel titles. **A.** CLCA1 (NM_001285) mutation distribution in non-metastatic (left, somatic mutation rate 1.8%) and metastatic (right, 21.1%) pNETs. The linear map highlights the CLCA_N, vWFA, hCaGC, and DUF1973 domains, showing a single in-frame insertion in non-metastatic tumors versus multiple in-frame and frameshift insertions and nonsense mutations concentrated toward the C-terminal region in metastatic tumors. **B.** RYR1 (NM_000540) mutation distribution in non-metastatic (left, 3.6%) and metastatic (right, 21.1%) pNETs. The full-length ryanodine receptor channel is shown with annotated channel and regulatory domains. Non-metastatic tumors harbor a small number of non-sense, in-frame insertion, and frameshift insertion events restricted between SPRY and RYDR_ITPR domains, whereas metastatic tumors exhibit a higher burden of missense, frameshift insertion, and nonsense mutations distributed heterogeneously across the domains. **C.** CEACAM5 (NM_004363) mutation distribution in non-metastatic (left, 5.4%) and metastatic (right, 36.8%) pNETs. The schematic shows the Ig-like CEACAM domains (e.g., Ig_CEACAM_D1 and Ig_CEACAM_D4) and C-terminal segments. Non-metastatic tumors contain infrequent missense and frameshift insertions, whereas metastatic tumors show a markedly higher frequency of missense and nonsense substitutions along the extracellular domain. **D.** MYO3B (NM_138995) mutation distribution in non-metastatic (left, 12.5%) and metastatic (right, 36.8%) pNETs. The schematic shows mutations in different MYO3B domains and C-terminal segments. Non-metastatic tumors contain frequent frameshift insertions, whereas metastatic tumors show in frame insertions.

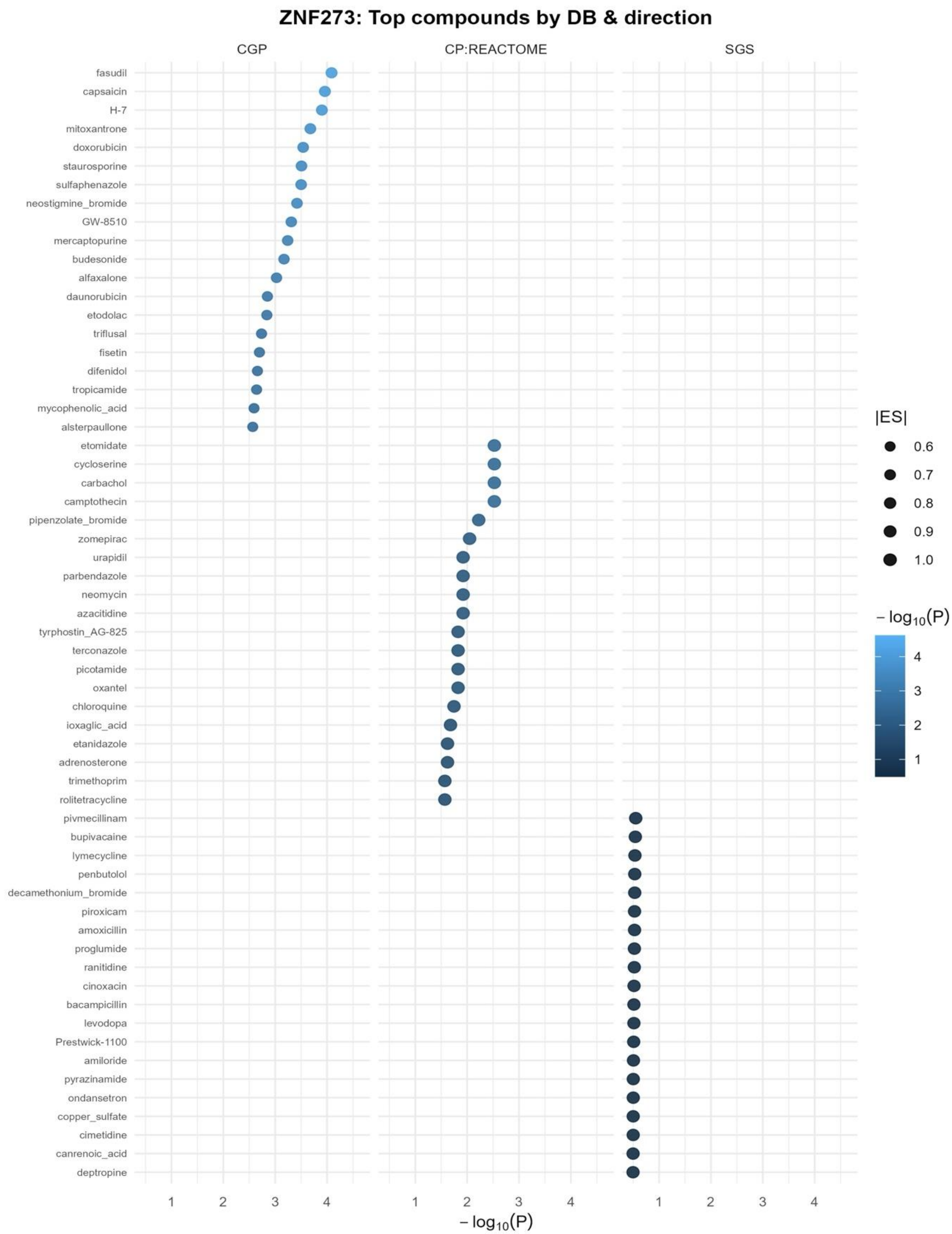


**Supplementary Figure 7. Gene2Drug-predicted compounds targeting ZNF273-associated transcriptional programs.** Bubble plot summarizing small-molecule compounds predicted by Gene2Drug to significantly counteract the gene-expression signature centered on ZNF273 in

metastatic pNETs. The y-axis lists individual compounds, and the x-axis shows the statistical significance of their enrichment, expressed as $-\log_{10}(p\text{-value})$. Points are grouped by annotation database used for the enrichment analysis: CGP (cancer gene perturbation signatures), CP:REACTOME (Reactome pathway-based signatures), and SGS (single-gene signatures). Each bubble represents a compound-database pair, bubble size encodes the absolute enrichment score (ES), and color intensity reflects $-\log_{10}(P)$, with darker shades indicating more significant associations.

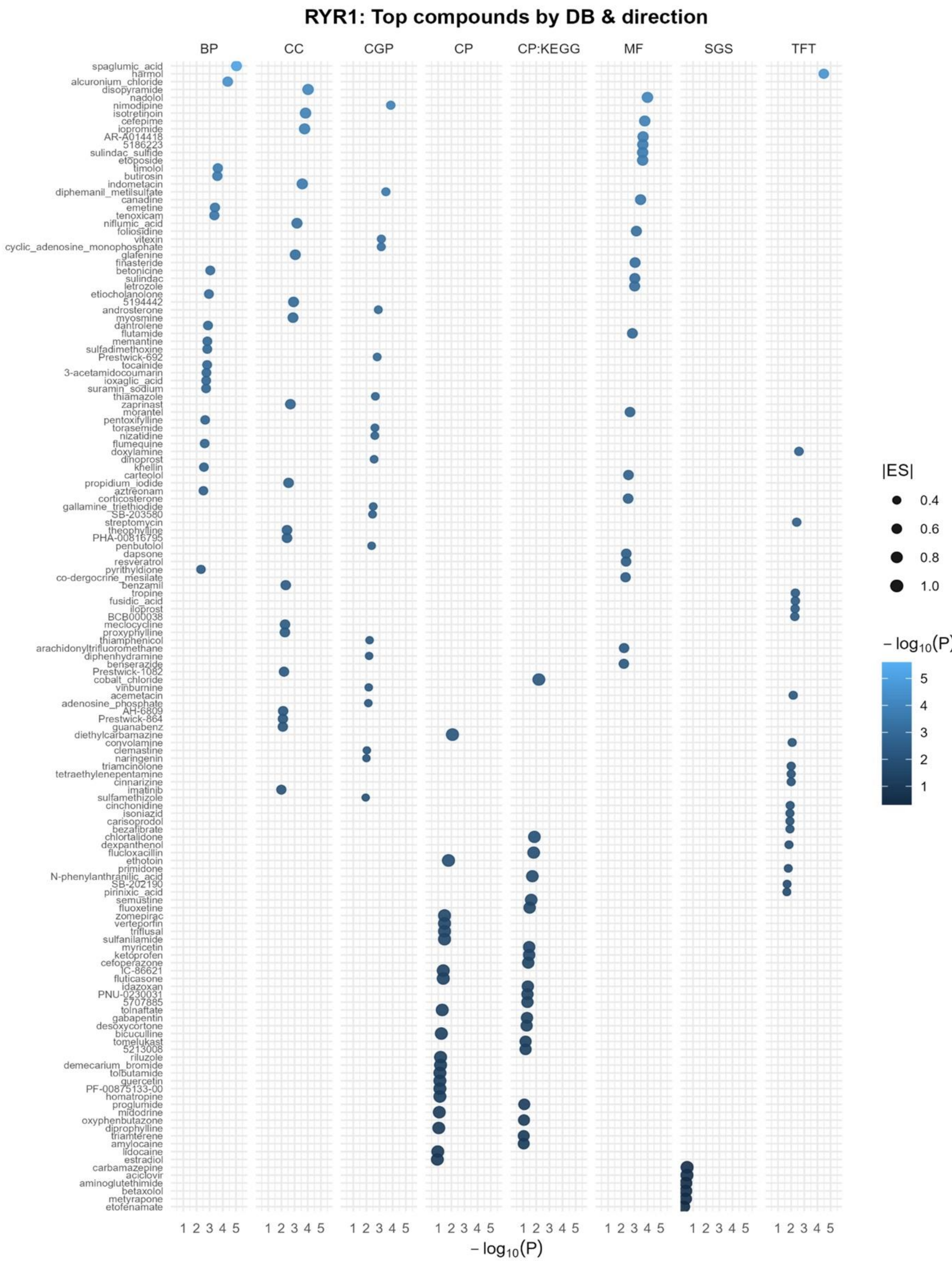


**Supplementary Figure 8. Gene2Drug-predicted compounds targeting RYR1-associated transcriptional programs.** Bubble plot summarizing small-molecule compounds predicted by

Gene2Drug to significantly counteract the RYR1-centered gene-expression signature in metastatic pNETs. The y-axis lists individual compounds, while the x-axis shows $-\log_{10}$(*p*-value) for the enrichment of each compound's perturbational profile. Bubbles are grouped into vertical columns according to the annotation database used: Gene Ontology Biological Process (BP), Cellular Component (CC), cancer gene perturbation signatures (CGP), Canonical Pathways (CP), KEGG pathways (CP:KEGG), Molecular Function (MF), single-gene signatures (SGS), and transcription-factor-target sets (TFT). Each bubble represents a compound-database pair; bubble size encodes the absolute enrichment score (ES), and color intensity reflects statistical significance ($-\log_{10}(P)$), with darker shades indicating more significant associations.

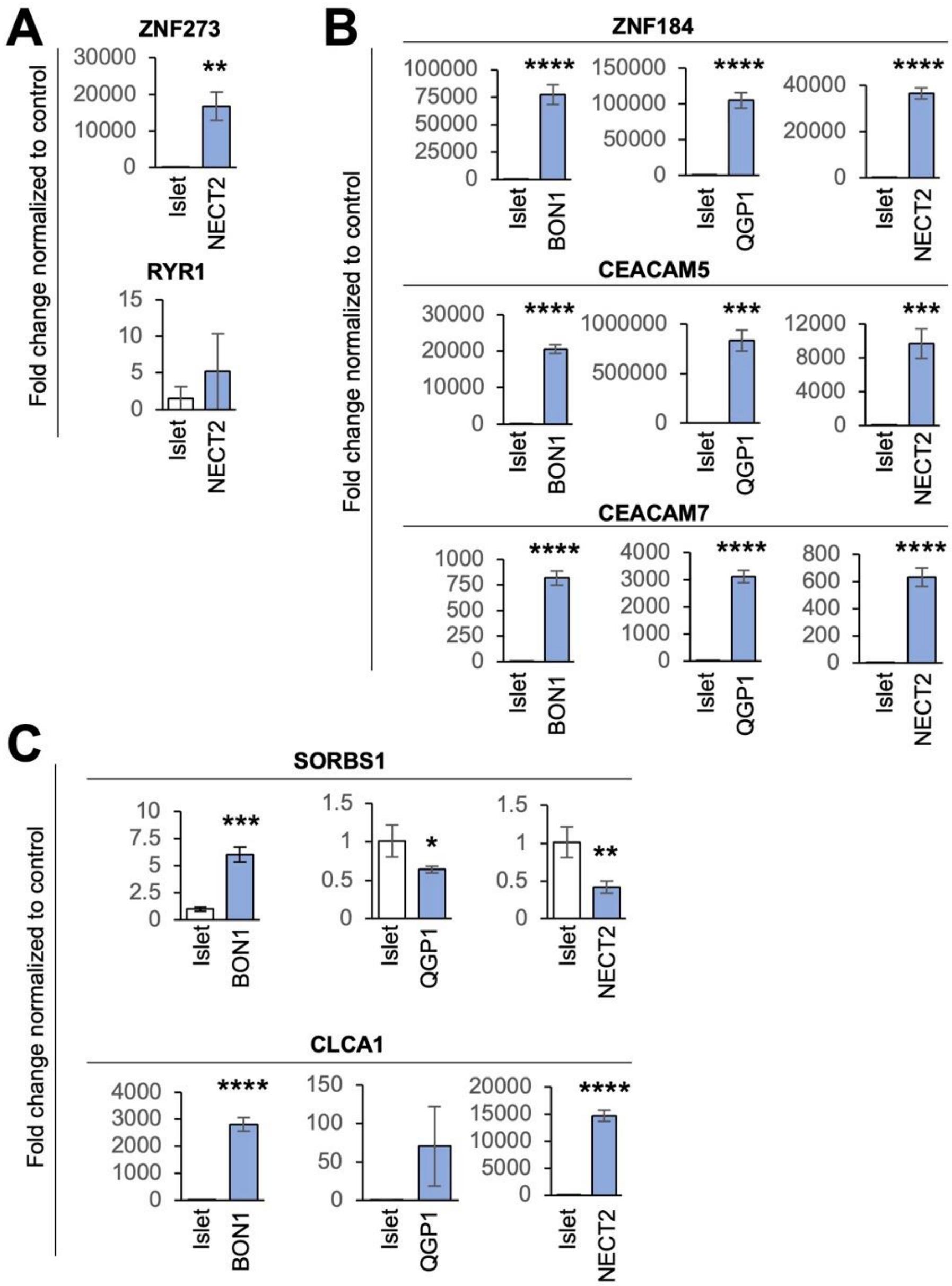


**Supplementary Figure 9. Real-time RT-qPCR validation of candidate transcripts in pancreatic neuroendocrine tumor cell lines relative to human islets.** Relative mRNA expression is shown as fold change normalized to the islet control. **A.** ZNF273 and RYR1 expression in NECT2 cells compared with islets. **B.** Upregulation of ZNF184, CEACAM5, and CEACAM7 in BON1, QGP1, and NECT2 cells compared with islets. **C.** Differential expressions of SORBS1 and CLCA1 in BON1, QGP1, and NECT2 cells compared with islets. Data are

presented as mean with standard deviation as shown. Statistical significance is indicated in the plots with asterisks (*, $P < 0.05$; **, $P < 0.01$; ***, $P < 0.001$ and ****, $P < 0.0001$).

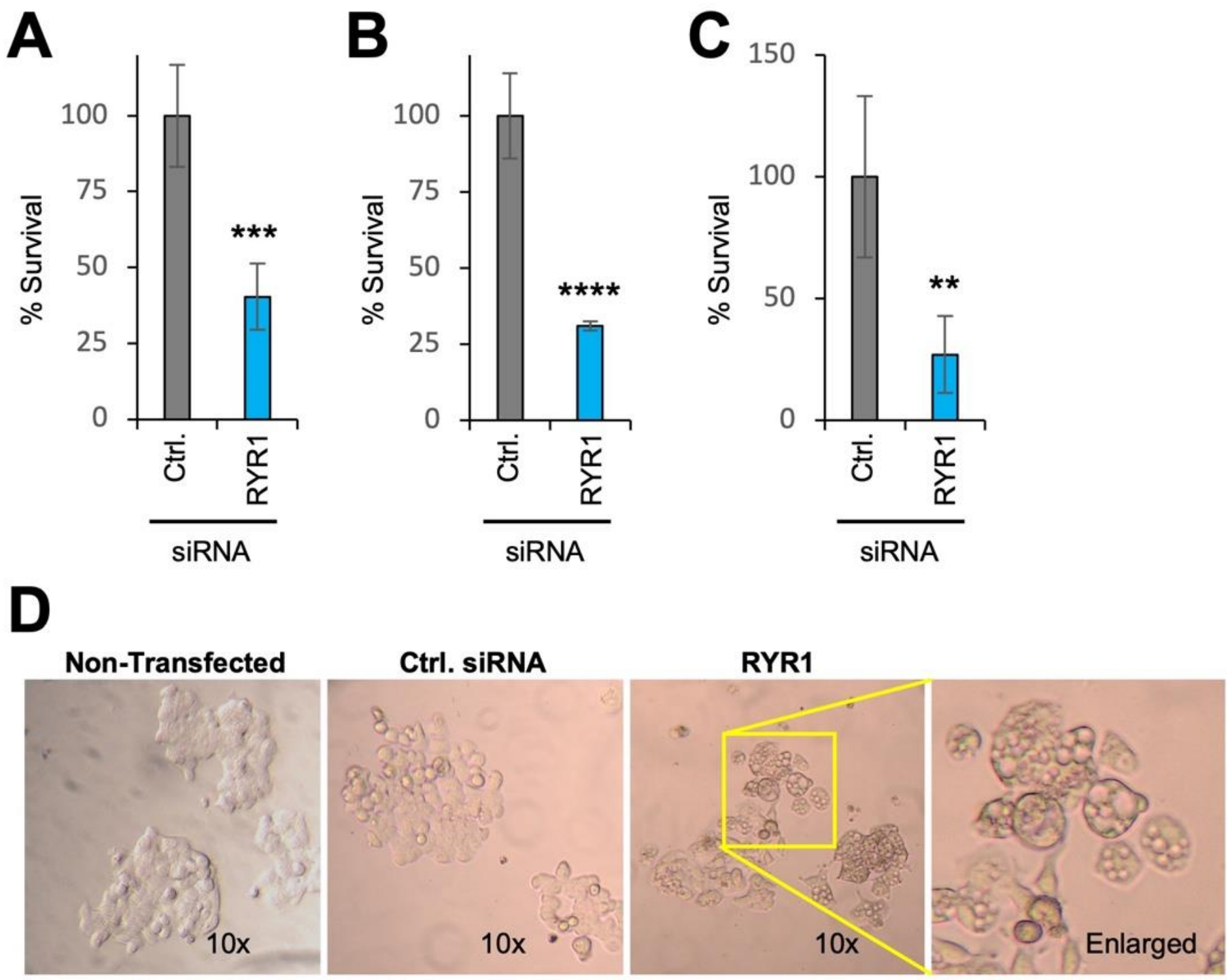


**Supplementary Figure 10. RYR1 silencing reduces BON1 cell viability and induces prominent vacuolization.** BON1 cells were transfected with control or RYR1-targeting siRNA and assayed for percent survival relative to control by MTT assay. **A.** 5-Day survival following RYR1 silencing using a double-dose siRNA regimen. **B.** 7-Day survival following RYR1 silencing using a single-dose siRNA regimen. **C.** 7-Day survival following RYR1 silencing using a double-dose siRNA regimen. **D.** Representative bright-field images (10x) of BON1 cells that were non-transfected or transfected with control siRNA or RYR1 siRNA and imaged at day 5 after transfection with single-dose siRNA. RYR1 siRNA treated cells display marked cytoplasmic vacuolization, the boxed region is shown enlarged at right. Bar graphs show mean ± standard deviation. Statistical significance is indicated in the plots with asterisks (**, *P* <0.01; ***, *P* <0.001 and ****, *P* <0.0001).

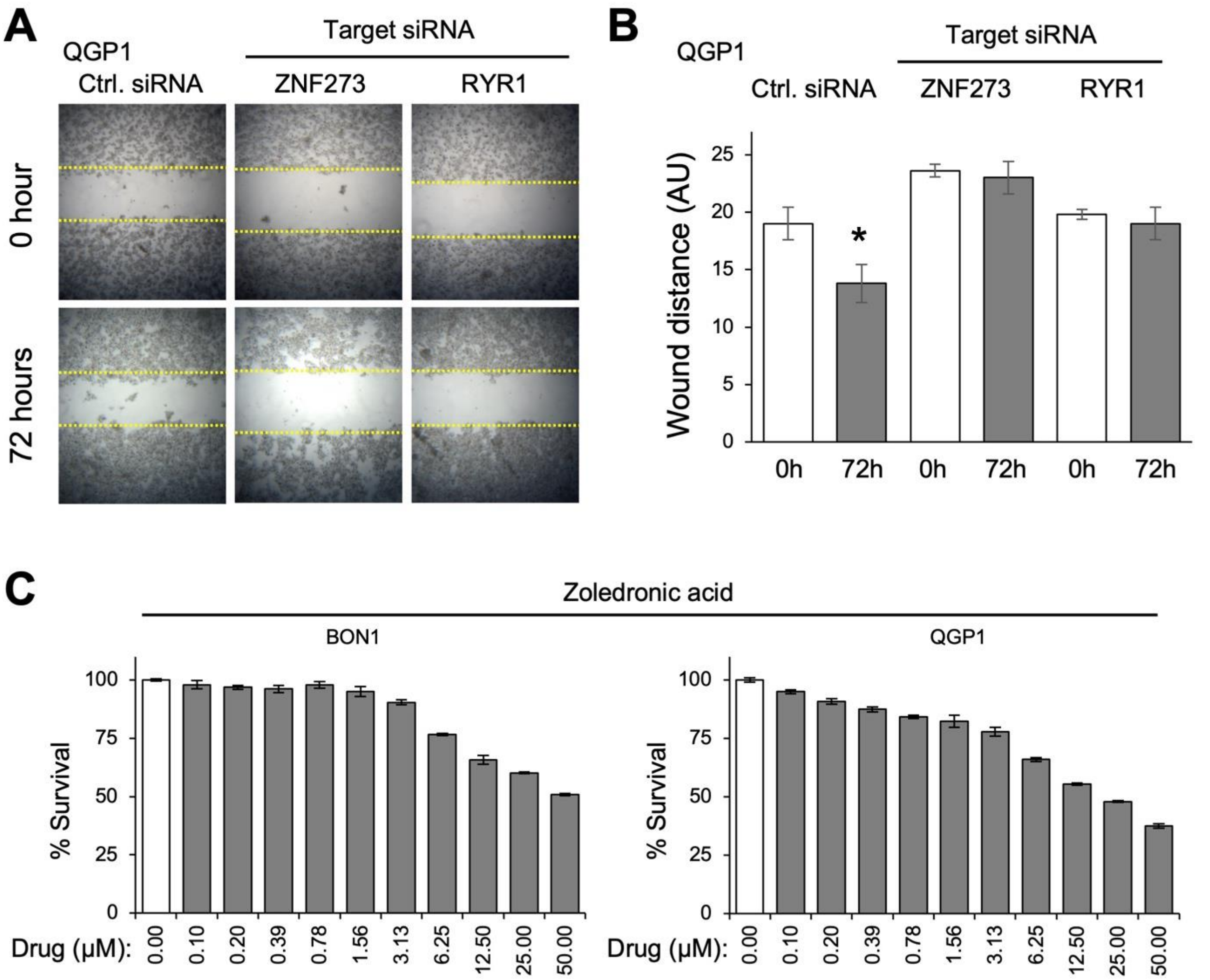


**Supplementary Figure 11. ZNF273 and RYR1 knockdown alters QGP1 wound closure, and zoledronic acid shows limited cytotoxic activity in pNET cell lines. A.** Representative wound-healing (scratch) assay images of QGP1 cells transfected with control siRNA or siRNAs targeting ZNF273 or RYR1. Wounds were generated at the time of transfection (0h) and re-imaged at 72h at the same field. The dashed lines mark wound edges. **B.** Quantification of wound distance at 0h and 72h for each condition. **C.** Dose-response viability analysis of BON1 and QGP1 cells treated with zoledronic acid for 72h. Cell survival is expressed as percent survival relative to vehicle control across the indicated drug concentrations (µM) and measured by MTT assay. Bar graphs show mean ± standard deviation. Statistical significance is indicated in the plots with asterisks (*, $P < 0.05$). AU, arbitrary unit.

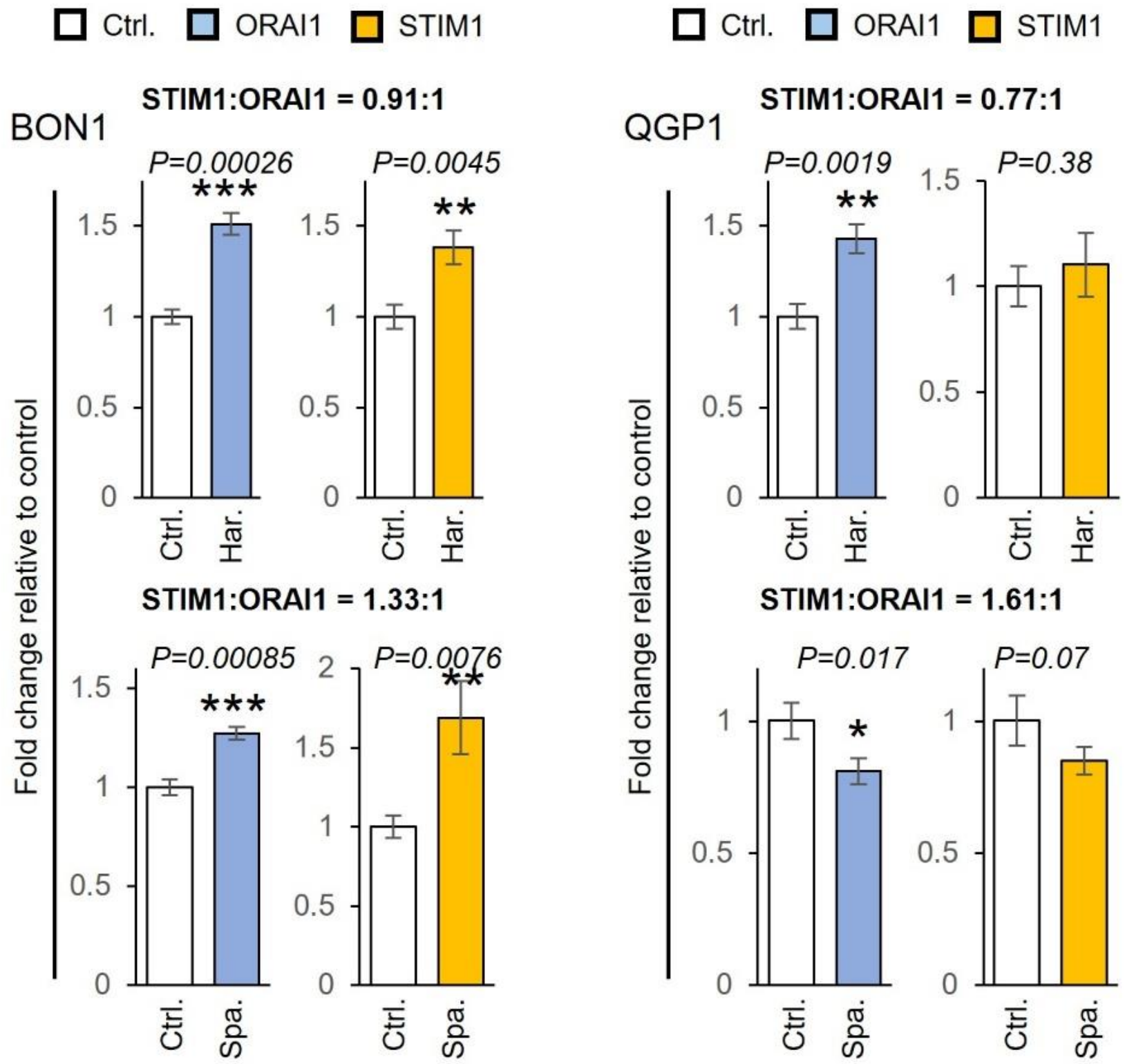


**Supplementary Figure 12. Real-time RT-qPCR analysis of calcium signaling associated transcripts in pancreatic neuroendocrine tumor cell lines BON1 and QGP1.** Relative mRNA expression is shown as fold change normalized to the control. ORAI1 and STIM1 expressions in BON1 cell lines treated with predicted drugs Har and Spa (left panel**).** ORAI1 and STIM1 expressions in QGP1 cell lines treated with predicted drugs Har and Spa (right panel**).** Data are presented as mean with standard deviation as shown. Student's t-test was performed for the comparison. Statistical significance is indicated in the plots with asterisks (*, P <0.05; **, P <0.01; and ***, P <0.001). Ctrl., control; Har, harmol; Spa, spaglumic acid.

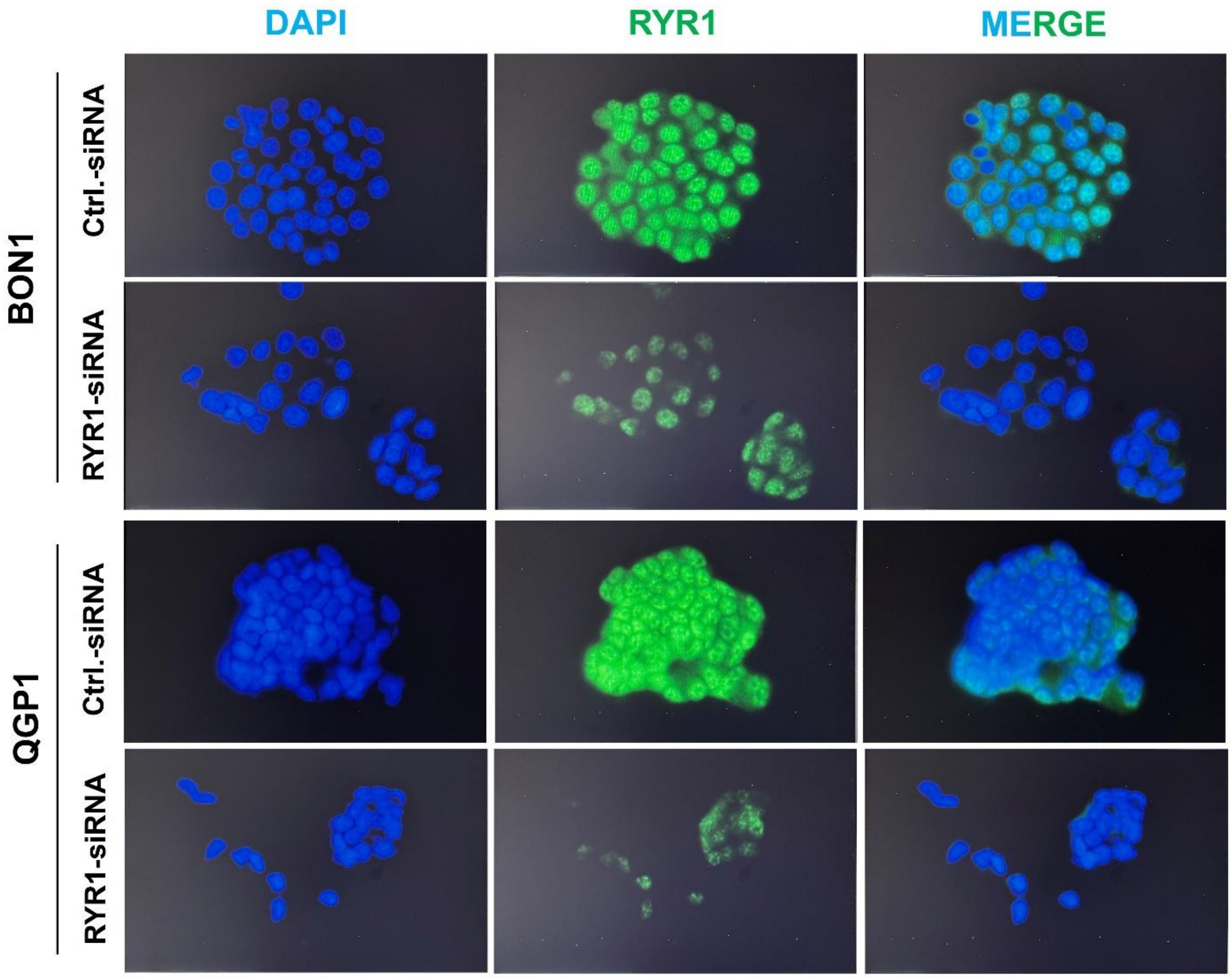


**Supplementary Figure 13. Immunofluorescence analysis of RYR1 silencing in pancreatic neuroendocrine tumor cell lines BON1 and QGP1.** Representative fluorescence microscopy images showing RYR1 expression and subcellular localization. RYR1 was detected using a rabbit anti-RYR1 primary antibody at a 1:200 dilution, followed by an anti-rabbit secondary antibody and is shown in green. Nuclei were counterstained with DAPI and are shown in blue. Original magnification: X400.

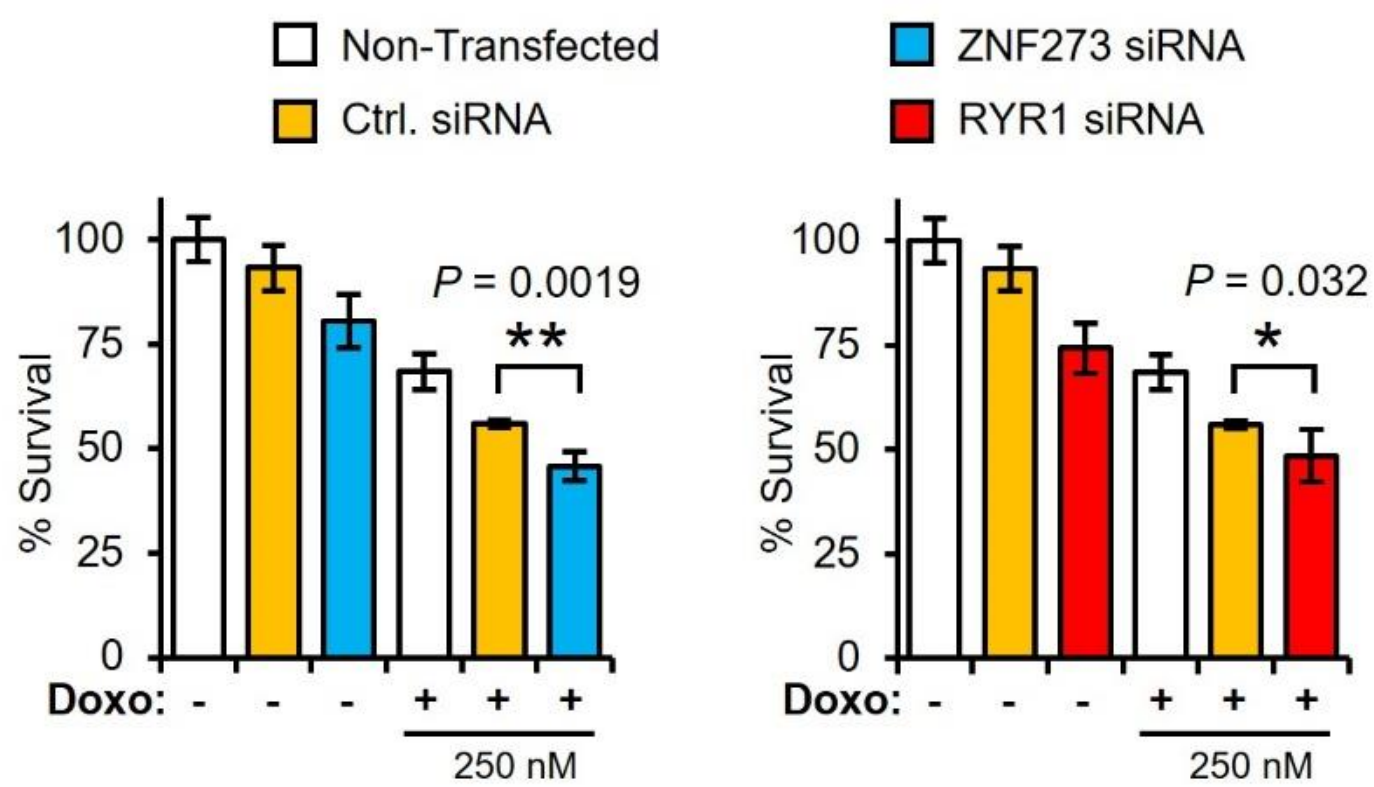


**Supplementary Figure 14. Sensitivity to doxorubicin (Doxo) upon silencing of ZNF273 or RYR1 in QGP1 pancreatic neuroendocrine tumor cell line.** Dose-response viability analysis of QGP1 cells treated with Doxo for 72h after silencing with ZNF273 targeted siRNA (left panel) or RYR1 targeted siRNA for 72h. Cell survival is expressed as percent survival relative to vehicle control with indicated drug concentration and measured by MTT assay. Data are presented as mean with standard deviation as shown. Student's t-test was performed for the comparison. Statistical significance is indicated in the plots with asterisks (*, P <0.05 and **, P <0.01). Ctrl., control; Doxo, doxorubicin.

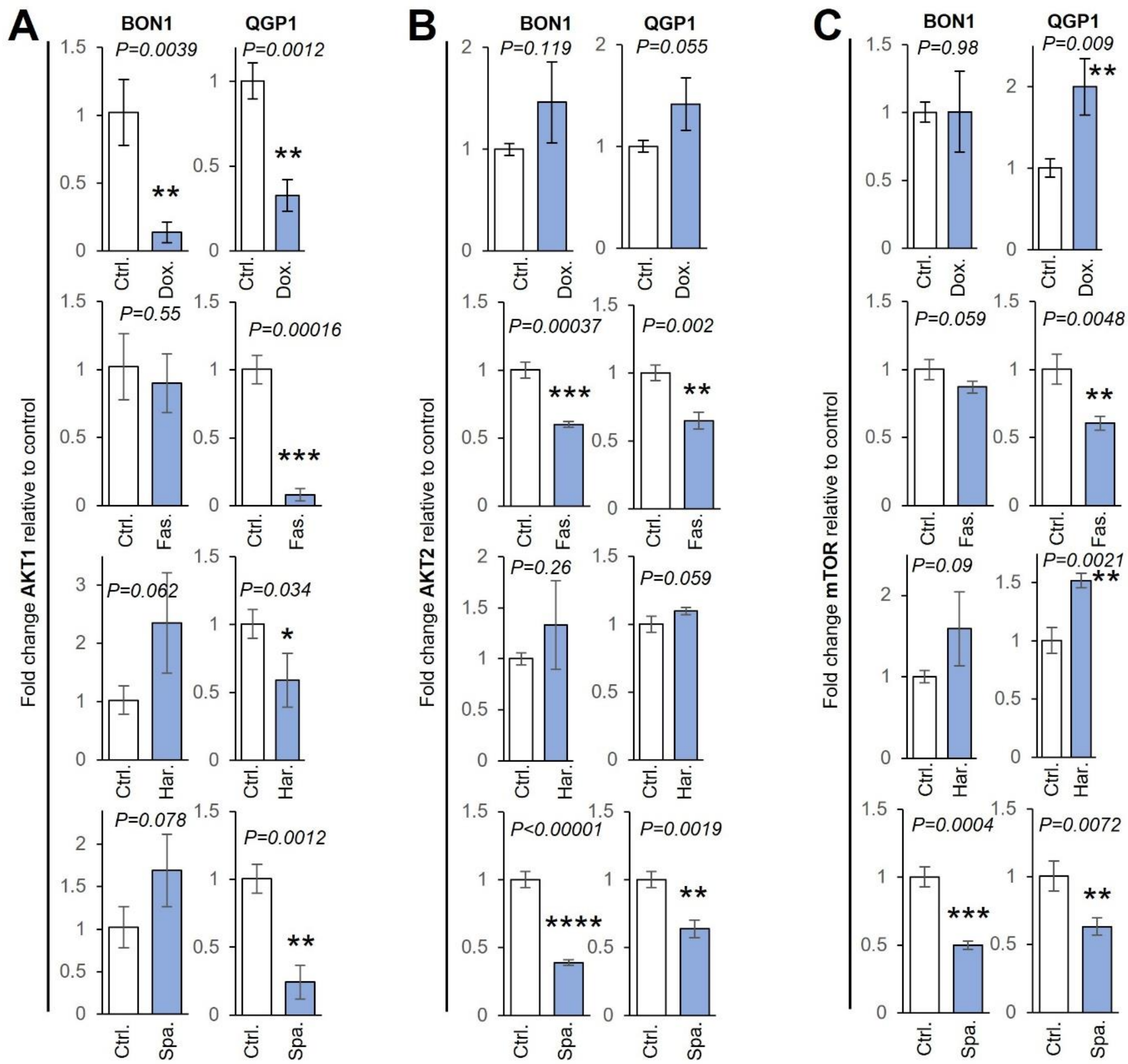


**Supplementary Figure 15. Real-time RT-qPCR analysis of proliferation pathway transcripts in pancreatic neuroendocrine tumor cell lines BON1 and QGP1.** Relative mRNA expression is shown as fold change normalized to the control. **A.** AKT1 expression in BON1 and QGP1 cell lines treated with predicted drugs. **B.** AKT2 expression in BON1 and QGP1 cell lines treated with predicted drugs. **C.** mTOR expression in BON1 and QGP1 cell lines treated with predicted drugs. Data are presented as mean with standard deviation as shown. Student's t-test was performed for the comparison. Statistical significance is indicated in the plots with asterisks (*, P <0.05; **, P <0.01; ***, P <0.001 and ****, P <0.0001). Ctrl., control; Dox, doxorubicin; Fas, fasudil; Har, harmol; Spa, spaglumic acid.

**References:**

[1] Uddin MH, Al-Hallak MN, Khan HY, Aboukameel A, Li Y, Bannoura SF, Dyson G, Kim S, Mzannar Y, Azar I, Odisho T, Mohamed A, Landesman Y, Kim S, Beydoun R, Mohammad RM, Philip PA, Shields AF, Azmi AS. Molecular analysis of XPO1 inhibitor and gemcitabine-nab-paclitaxel combination in KPC pancreatic cancer mouse model. Clin Transl Med. 2023 Dec;13(12):e1513. doi: 10.1002/ctm2.1513.

[2] Uddin MH, Li Y, Khan HY, Muqbil I, Aboukameel A, Sexton RE, Reddy S, Landesman Y, Kashyap T, Azmi AS, Heath EI. Nuclear Export Inhibitor KPT-8602 Synergizes with PARP Inhibitors in Escalating Apoptosis in Castration Resistant Cancer Cells. Int J Mol Sci. 2021 Jun 22;22(13):6676. doi: 10.3390/ijms22136676.
